# Autophagy-enhanced nanosonosensitizer mediated sonodynamic therapy for post-myocardial infarction neuromodulation and arrhythmia prevention

**DOI:** 10.7150/thno.103780

**Published:** 2025-01-13

**Authors:** Haoyuan Hu, Songyun Wang, Qian Li, Jiahui Zhao, Yida Pang, Jiale Wang, Huijun Wu, Xinqi Wang, Ye Cheng, Mengran Yu, Xinyue Yin, Yan Zhang, Lilei Yu, Yao Sun, Hong Jiang

**Affiliations:** 1 Department of Cardiology, Renmin Hospital of Wuhan University; Cardiac Autonomic Nervous System Research Center of Wuhan University; Cardiovascular Research Institute, Wuhan University; Hubei Key Laboratory of Cardiology, Wuhan, China.; 2 State Key Laboratory of Green Pesticide, International Joint Research Center for Intelligent Biosensor Technology and Health College of Chemistry, Central China Normal University, China.

**Keywords:** Sonodynamic therapy, Nanosonosensitizer, Autophagy, Neuromodulation, Myocardial infarction, Arrhythmia

## Abstract

**Rationale:** Sympathetic hyperactivation and neuroinflammation are the main triggers of malignant ventricular arrhythmias (VAs) after myocardial infarction (MI). Previous studies proved that photothermal therapy (PTT) and photodynamic therapy (PDT) could reduce MI-induced VAs by inhibiting neuroinflammation. However, the limited penetration depth and potential phototoxicity of phototherapy impose constraints on its further application. As a treatment strategy derived from phototherapy, sonodynamic therapy (SDT) offers exceptional advantages, including excellent penetration capability, temporal-spatial controllability, superior efficacy and minimal side effects. Therefore, it is worthwhile to investigate the effects of sonodynamic modulation on neuroinflammation and arrhythmia prevention.

**Methods:** We designed a long-wavelength emissive sonosensitizer (named **BBTD-TPA**) based on donor-acceptor-donor scaffold. Subsequently, the compound was encapsulated in DSPE-PEG5000 to form **BBTD-TPA** nanoparticles (NPs). *In vitro* experiments were conducted to determine the optimal concentration of **BBTD-TPA** NPs-mediated SDT and to verify the effects and pathways on autophagy in BV2 cells. The distribution and metabolism of **BBTD-TPA** NPs *in vivo* were assessed by NIR-II fluorescence imaging. Finally, *in vivo* studies were performed to assess the effect of **BBTD-TPA** NPs-mediated SDT on post-MI sympathetic neuroinflammation and the occurrence of VAs.

**Results:** *In vitro* studies demonstrated that **BBTD-TPA** NPs combined with LIFU could promote microglial autophagy via the ROS-AMPK-mTOR pathway. **BBTD-TPA** NPs were further microinjected into the paraventricular nucleus (PVN), real-time NIR-II fluorescence imaging showed that **BBTD-TPA** NPs could remain in the PVN for up to 12 h and be metabolized through the liver and kidney. Further *in vivo* results verified that **BBTD-TPA** NPs-mediated SDT could inhibit sympathetic nervous activity, and inflammatory responses, thus preventing MI-induced VAs.

**Conclusion: BBTD-TPA** NPs-mediated SDT can promote microglial autophagy and inhibit sympathetic neuroinflammation, thus reducing MI-induced VAs. The current research may inspire a novel strategy for neuromodulation and arrhythmia prevention, providing broader prospects for clinical translation of nanomedical technology.

## Introduction

Ventricular arrhythmia (VA) is considered the predominant cause of sudden death following myocardial infarction (MI) 1-3. The hyperactivation of sympathetic nerves and neuroinflammation after MI are closely related to the occurrence of VAs 4-6. Previous studies have demonstrated that acute MI might result in microglial activation and sympathetic neural activation in the paraventricular nucleus (PVN) and left stellate ganglion (LSG), and these two might facilitate each other, thus forming a vicious cycle 7-9. Traditional clinical interventions include LSG resection and ablation for the treatment of VAs 10,11. However, the clinical used LSG ablation might cause chest pain, Horner's syndrome and other adverse reactions, which have limited its further application 12. Therefore, it is imperative to investigate a safer and more efficient intervention strategy.

Recent literature indicated that light-emitting diode (LED), photothermal therapy (PTT), photodynamic therapy (PDT), and low-intensity focused ultrasound (LIFU) might attenuate sympathetic and microglial activation in the PVN or LSG, thus preventing VAs 13-16. However, hindered by the low tissue penetration and phototoxicity of phototherapy and the deficient spatial resolution of LIFU, precise positioning cannot be achieved, and the therapeutic effect needs to be further improved. Sonodynamic therapy (SDT), as a treatment method with deeper penetration depth, precise tissue positioning, and fewer side effects, has been applied for the treatment of tumors, infections and atherosclerosis 17-22. In the presence of ultrasound, sonosensitizers can convert local oxygen molecules into reactive oxygen species (ROS), thus inducing cellular apoptosis or autophagy 23-25. Recently, Yang *et al.* demonstrated that non-lethal SDT could inhibit the inflammatory response of atherosclerotic plaques by enhancing macrophage autophagy 26. Moreover, previous studies demonstrated that autophagy played a crucial role in regulating neuroinflammation and promoting microglial autophagy might inhibit neuroinflammation 27,28. Therefore, it is feasible to modulate neuroinflammation by autophagy-enhanced SDT.

**Figure 1:**
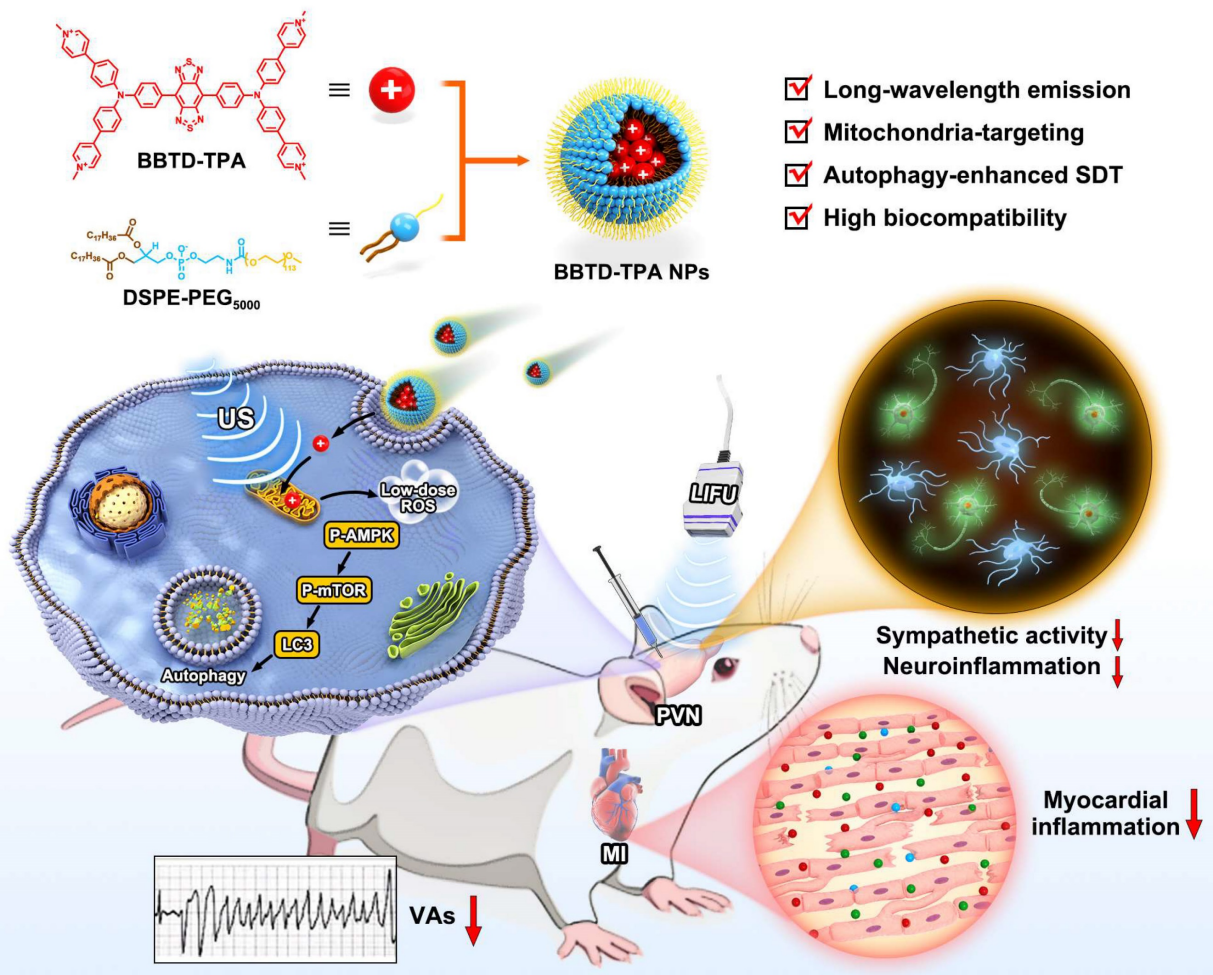
Schematic diagram of the synthetic procedure of **BBTD-TPA** NPs and the mechanism of sonodynamic modulation of neuroinflammation to prevent post-MI VAs.

Recently, organic sonosensitizers have been highlighted in the biomedicine due to their high biocompatibility and definite chemical structure 29,30. Moreover, incorporating an organic sonosensitizer into the amphiphilic copolymer (such as DSPE-PEG) to form nanosonosensitizer and offering organic sonosensitizer with sub-organelle targeting ability are no doubt to significantly enhance SDT efficiency 31-34. However, most of the existing organic sonosensitizers are short-wavelength emission, rendering them unsuitable for deep-tissue imaging 35. In light of these considerations, herein, we designed a long-wavelength emissive sonosensitizer based on donor-acceptor-donor scaffold (named **BBTD-TPA**), comprising benzo[1,2-c:4,5c′]bis[1,2,5]thiadiazole (BBTD) unit as the acceptor and triphenylamine (TPA) unit as the donor. After encapsulation in DSPE-PEG5000 to form nanoparticles (NPs), **BBTD-TPA** NPs demonstrated mitochondria targeting, NIR-II fluorescence imaging properties, superior penetration depth and favorable biocompatibility. Furthermore, we explore the role and mechanism of **BBTD-TPA** NPs-mediated autophagy-enhanced sonodynamic modulation of neuroinflammation in preventing MI-induced VAs (**Figure 1**). To the best of our knowledge, this is the first attempt of SDT for neuroinflammatory modulation and cardiac arrythmia prevention in a MI model.

## Materials and methods

More experimental details of methods and materials are available in the **Supplementary Material**.

### Synthesis and characterization of BBTD-TPA NPs

**BBTD-TPA** was synthesized by the methods mentioned in the **Supplementary Material**. One milliliter of **BBTD-TPA** solution (1 mg mL^-1^ in acetonitrile) and 9 mL of DSPE-PEG5000 solution (1 mg mL^-1^ in ddH_2_O) were mixed by an ultrasonic homogenizer for 10 min. Then, the acetonitrile was removed with nitrogen gas, and the mixture was centrifuged at 3000 rpm for 10 min using a 50 kDa ultrafiltration centrifugal tube. The transmission electron microscopy (TEM) images of **BBTD-TPA** NPs were obtained by a JEM2100F microscope (200 kV). Dynamic light scattering (DLS) of **BBTD-TPA** NPs was recorded by a Malvern Zetasizer Nano ZS90. All UV-vis absorption curves were measured by a Varian Cary 50 UV-vis spectrophotometer.

### *In vitro* sonostability test

**BBTD-TPA** NPs (20 μM) were continuously subjected to ultrasound (US) irradiation for 30 min (1.0 MHz, 0.5 W cm^-2^). The absorbance was measured every 5 min by using a microplate reader.

### Determination of ROS levels *in vitro*

The ROS generation efficacy of **BBTD-TPA** NPs was estimated by the commonly used ROS indicator dichlorofluorescein diacetate (DCFH-DA). DCFH-DA (10 mM in DMF, 100 μL) was mixed with NaOH (10 mM in ddH_2_O, 0.5 μL) followed by stirring at room temperature for 30 min to hydrolyze into DCF. Activated DCFH-DA (50 µM) was added to an aqueous solution of **BBTD-TPA** NPs (20 µM) and PBS, and then the mixture was exposed to US irradiation for various time intervals. The fluorescence spectra of DCF (λex= 488 nm, λem= 525 nm) induced by **BBTD-TPA** NPs were recorded. 1,3-diphenylisobenzofuran (DPBF) (1.2 μL, 2 mg mL^-1^) dissolved in DMSO was added to **BBTD-TPA** NPs solution (20 µM, 600 μL), followed by exposure to US irradiation (1 MHz, 0.5 W cm^-2^) for various durations. The declining UV‒Vis absorption at 420 nm indicated the production of ^1^O_2_.

### Cytotoxicity assay

The BV2 cells were cultured in MEM with 10% FBS and 1% penicillin-streptomycin-gentamicin solution, and incubated in an incubator at 37 °C with a continuous supply of 5% CO₂. The detected BV2 cells were seeded in a 96-well plate (1 × 10^4^ cells/well) and cultured for 12 h. Subsequently, the medium in each well was replaced with fresh MEM and fresh MEM containing **BBTD-TPA** NPs at various concentrations. After 12 h of incubation, the culture medium was replaced with fresh MEM, and BV2 cells were treated with or without US irradiation (0.5 W cm^-2^, 1 MHz, 50% duty cycle, 5 min). After an additional 12 hours of incubation, cell viability was recorded using standard CCK-8 methods.

### Flow cytometry analysis

BV2 cells were plated in 6-well plates for 12 h at a cell density of 5 × 10^5^ cells mL^-1^. Then the four groups of control, US, **BBTD-TPA** NPs, and SDT were set up to treat the BV2 cells for 12 h. For DCFH-DA staining, the BV2 cells were incubated at 37 ℃ for 30 min to ensure full dye internalization. Moreover, the intracellular fluorescence intensity was also quantified by flow cytometry analysis (Beckman Cytoflex, Suzhou, China), and the data were analyzed using FlowJo V10.

### Western blot analysis

To extract proteins, RIPA lysis buffer mixed with PMSF and Cocktail was added to the cell/tissue samples. The proteins were then separated by SDS-PAGE, transferred to a PVDF membrane, and fixed in 5% BSA solution. Subsequently, the membrane was then incubated with primary antibody anti-LC3B (A19665, ABclonal, China), anti-p62 (A19700, ABclonal, China), anti-AMPK (AF6423, Affinity, USA), anti-Phospho-AMPK (GB114323, Servicebio, China), anti-mTOR (GB11405, Servicebio, China), anti-Phospho-mTOR (GB114489, Servicebio, China), anti-IL-1β (GB11113, Servicebio, China), anti-IL18 (A23076, ABclonal, China), anti-TNF-α (GB113968, Servicebio, China), anti-GAPDH (GB15004, Servicebio, China) and anti-β-acitn (AC038, ABclonal, China) overnight at 4°C, followed by a secondary antibody for 2 h. The images were acquired using ECL chemiluminescence and analyzed by Image J software.

### Animal preparation

Forty SD rats were included in the *in vivo* experiment. The experimental procedure was approved by the Animal Ethics Committee of Wuhan University (approval number: 20230510A) and was conducted in accordance with the NIH guidelines. The rats were anesthetized using 1.5% isoflurane inhalation before **BBTD-TPA** NPs injection and electrophysiological experiment *in vivo*.

### BBTD-TPA NPs injection into the PVN and LIFU irradiation

The PVN was located 0.3 mm lateral, 1.8 mm posterior, and 7.9 mm inferior to the skull fontanelle (**Figure S1**). After anesthesia, **BBTD-TPA** NPs (400 μM, 5 μL) were injected into the PVN on each side in the SDT group by using a stereotaxic instrument, and saline was injected into the remain three groups. LIFU (1.0 MHz, 2.0 W cm^-2^) irradiation was conducted at the skull surface for 10 min. The representative graphs of ultrasound probe and LIFU irradiation are shown in **Figure S2**.

### *In vivo* and *ex vivo* NIR-II fluorescence imaging

After the injection of** BBTD-TPA** NPs (400 μM, 5 μL) into the PVN, the fluorescence images were obtained using NIR-II fluorescence imaging system (Suzhou NIR-Optics imaging system). After 12 h of local injection, rats were executed and various organs were removed to assess the distribution of **BBTD-TPA** NPs.

### Establishment of MI model

To make the MI model, ligation of the left anterior descending artery (LAD) with an opening chest at the third or fourth intercostal space was performed. Myocardial discoloration in the area of ischemia or ST-segment elevation and T-wave changes on the electrocardiogram (ECG) were used as criteria to confirm acute infarction 36.

### Heart rate variability (HRV) analysis

ECG were recorded with a PowerLab data acquisition system. HRV was determined by 5-min stable ECGs using LabChart software, including low frequency (LF, 0.25-0.75 Hz), high frequency (HF, 0.75-2.5 Hz), and LF/HF ratio 37.

### Recording of ventricular arrhythmia event

After LAD ligation, the occurrence of ventricular arrhythmia events was monitored with continuous ECG recording for 30 min. Ventricular premature beat (VPB) was defined as early-onset QRS waves. Ventricular tachycardia (VT) was defined as more than 3 continuous VPBs, of which those lasting longer than 30 seconds were considered sustained ventricular tachycardia (SVT) and those lasting less than 30 seconds were considered non-sustained ventricular tachycardia (nSVT). Ventricular fibrillation (VF) was defined as tachycardia that was morphologically unstable and unrecognizable on the ECG.

### Programmed electrophysiological stimulation

Eight paced beats (S1) set at a lower cycle length than the RR interval, followed by 1-3 additional stimuli (S2, S3, and S4) set at shorter intervals, were used to measure the inducibility of VA. 0 points, no VA induced; 1 point, non-sustained VA induced by three additional stimuli; 2 points, sustained VA induced by three additional stimuli; 3 points, non-sustained VA induced by two additional stimuli; 4 points, sustained VA induced by two additional stimuli; 5 points, non-sustained VA induced by one additional stimulus; 6 points, sustained VA induced by one additional stimulus; 7 points, VA induced during S1 stimulation; 8 points, cardiac arrest/various VAs before pacing 15.

### LSG neural activity recording

The LSG neural activity was recorded with a pair of silver electrodes hooked to the LSG and connected to an amplifier via a wire. The LSG electrical signal was acquired for 5 min by using a PowerLab system. LSG neural activity was characterized by two key parameters: amplitude and frequency 15. The ratios of frequency and amplitude with different time periods (post-LIFU, post-MI) to baseline status were used separately for all the groups to demonstrate the changes in neural activity.

### Immunofluorescence staining

After the experiment, rats were euthanized by 5% isoflurane inhalation, and brain tissue was taken for PVN localization sectioning. After fixation of frozen sections, immunofluorescence staining was performed using anti-TH (GB11181, Servicebio, China) to analyze central sympathetic activity. Microglial activity was analyzed using anti-Iba-1 (GB11181, Servicebio, China) staining. In addition, the levels of autophagy in the PVN were assessed using anti-LC3B (A19665, ABclonal, China) staining. The immunofluorescence staining images was handled using Image-Pro Plus 6.0 by an investigator blinded to the group information.

### Safety testing

The temperature of the tissue surrounding the PVN was measured using a thermal camera before and after ultrasound irradiation in the LIFU and SDT group. At the end of the experiment, the rats were sacrificed and the heart, liver, kidney, spleen and lungs were collected for H&E staining to observe and analyze the major organ injury in the experiment. Moreover, blood samples were obtained by puncture from the right ventricle to perform blood cell count and biochemical indicators detection.

### Statistical analysis

The data were presented as mean ± SEM or median with interquartile range. Two-way ANOVA was used to analyze the frequency and amplitude of LSG. Unpaired t-test was used for analyzing the temperature change of PVN. Nonparametric test was used to analyze the occurrence of VAs, arrhythmia score and QT interval. The rest of the data were analyzed through one-way ANOVA. All the data were analyzed and plotted on statistical graphs through GraphPad Prism 8.0 software (GraphPad Software, Inc., CA, USA). Statistical significance was defined as *P < 0.05*.

## Results and Discussion

### Preparation and characterization of BBTD-TPA NPs

**Figure 2:**
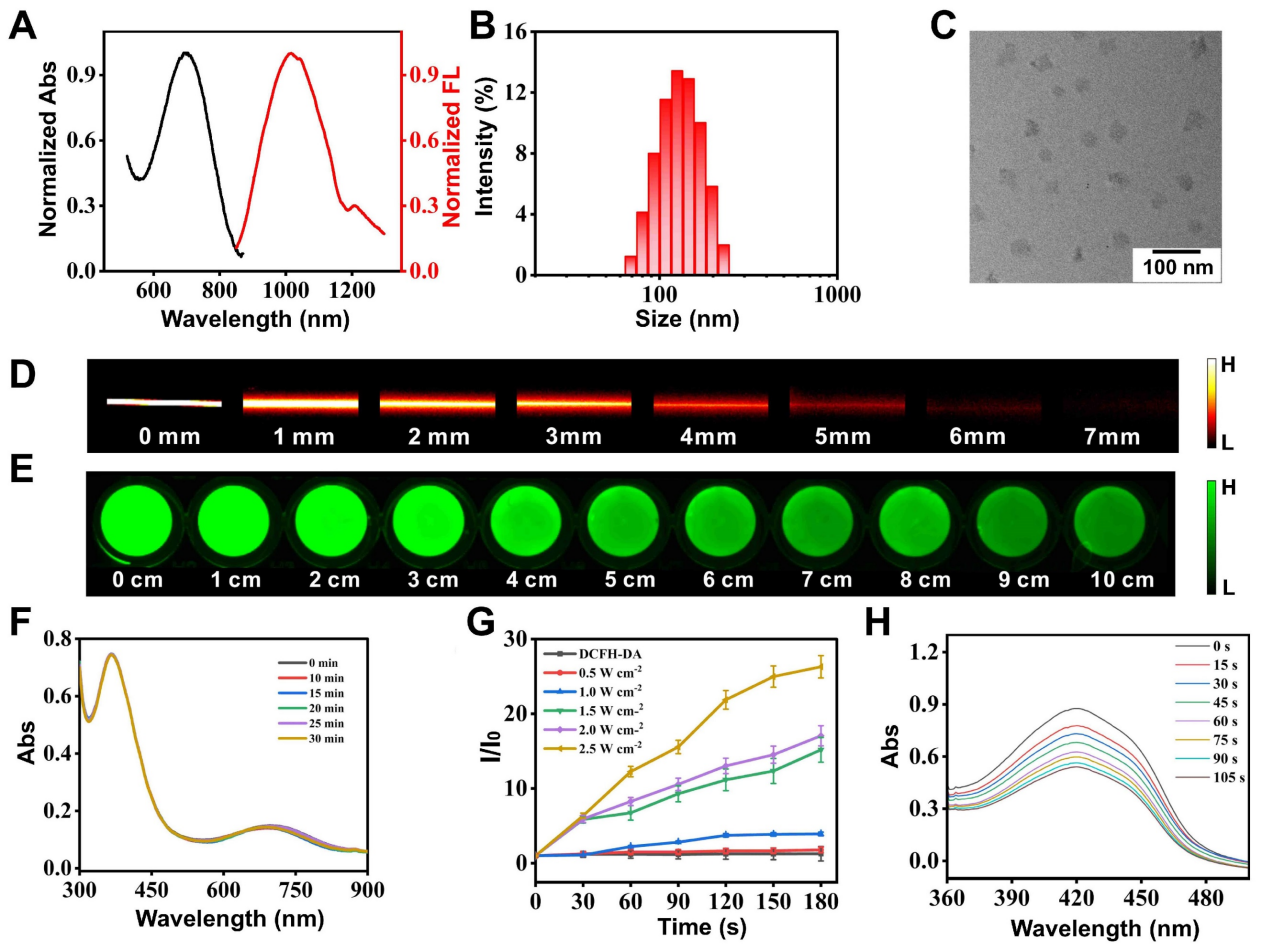
Characteristics of **BBTD-TPA** NPs. A) UV-vis and emission curves of **BBTD-TPA** NPs. B) Size distribution of** BBTD-TPA** NPs. C) TEM morphology of **BBTD-TPA** NPs. D) Fluorescence pictures of **BBTD-TPA** NPs encapsulated in capillaries in 0-7 mm 1% Intralipid. E) ROS imaging images by DCFH-DA probe staining after US irradiation of **BBTD-TPA** NPs placed on simulated tissues of different depths. F) UV-vis curves of **BBTD-TPA** NPs under US irradiation for 0-30 min. G) Comparison between the fluorescence intensity of DCFH-DA treatment with US irradiation for 0-2.5 W cm^-2^. H) UV‒vis curves of DPBF after coincubation with **BBTD-TPA** NPs under different US irradiation times.

The synthesis route and characterization of compound **BBTD-TPA** are shown in **Figure S3-S10**. **BBTD-TPA** NPs were obtained by encapsulating **BBTD-TPA** into DSPE-PEG5000 (encapsulation rate: 49.35%). First, the absorption peak of **BBTD-TPA** NPs was located at 678 nm, and the maximum fluorescence emission was in the second near infrared window (λ_em_ = 1000 nm, **Figure 2A**). Then, DLS result indicated that the size of **BBTD-TPA** NPs was approximately 125 nm (**Figure 2B**), and the uniform distribution of **BBTD-TPA** NPs in water could be observed by TEM (**Figure 2C**). Collectively, these results confirmed the successful constitution of **BBTD-TPA** NPs. Previous studies suggested that DSPE-PEG-coated nanoparticles could be disassembled in lysosomes to release the carried drugs 38,39. Based on this, we conducted an in vitro simulation of the acidic environment within lysosomes, examined the DLS and observed the distribution of **BBTD-TPA** NPs by TEM under different pH conditions. The DLS of **BBTD-TPA** NPs exhibited relative homogeneity in environments with near-neutral pH. Conversely, at pH 5.0, the size distribution of **BBTD-TPA** NPs exhibited significant heterogeneity, which varied considerably with incubation time (**Figure S11**). Similarly, **BBTD-TPA** was incubated in the solutions at pH 5.0 for 12 h. The TEM images demonstrated that the particle sizes of **BBTD-TPA** NPs exhibited significant variability at pH 5.0 (**Figure S12**). These results indicated that **BBTD-TPA** NPs may release **BBTD-TPA** in the acidic environment of cellular lysosomes. Next, to examine the fluorescence and ultrasound penetration depth of **BBTD-TPA** NPs *in vitro*, 1% intralipid was used to simulate biological tissues. As shown in **Figure 2D**, the fluorescence penetration depth of **BBTD-TPA** NPs was greater than 6 mm under 808 nm laser irradiation, which highlighted the advantages of NIR-II fluorescence technique for *in vivo* imaging. In addition, the penetration depth of US irradiation is reflected by the ROS production of **BBTD-TPA** NPs. As shown in **Figure 2E,** ROS production was still observed at a tissue depth of 10 cm, demonstrating that **BBTD-TPA** NPs in deeper tissues exceeding 10 cm are still capable of generating ROS under US irradiation, which facilitates sonodynamic modulation of neuroinflammation in deeper tissue. Furthermore, the stability of sonosensitizers is a vital aspect for their biomedical applications. As shown in **Figure 2F**, **BBTD-TPA** NPs were still able to maintain good stability after 30 min of US irradiation, as indicated by UV‒vis absorption spectrum. Then, the generation of ROS by **BBTD-TPA** NPs *in vitro* was investigated. As displayed in **Figure 2G** and **Figure S13**, ROS generation progressively increased with increasing US power. The fluorescence intensity of DCF was enhanced by 26-fold and 17-fold for 2.5 W cm^-2^ and 2.0 W cm^-2^ conditions, and by 15-fold, 4-fold, and 2-fold for 1.5 Wcm^-2^, 1.0 W cm^-2^, and 0.5 W cm^-2^, respectively. ROS production under low-power US irradiation was much less effective than that of high power, which confirms that US power has an effect on ROS production. Therefore, in this study, we adopted low-power US to generate trace amounts of ROS to perform a regulatory role. Moreover, the singlet oxygen (^1^O_2_) production ability of **BBTD-TPA** NPs was also detected by a DPBF probe. With prolonged US irradiation time, the absorbance at 420 nm continually decreased (**Figure 2H**). The results showed that **BBTD-TPA** NPs mainly produced ^1^O_2_. The satisfactory performance of **BBTD-TPA** NPs bodes well for their applications in biological fields.

### BBTD-TPA NPs-mediated SDT promoted microglial autophagy at cellular level

**Figure 3:**
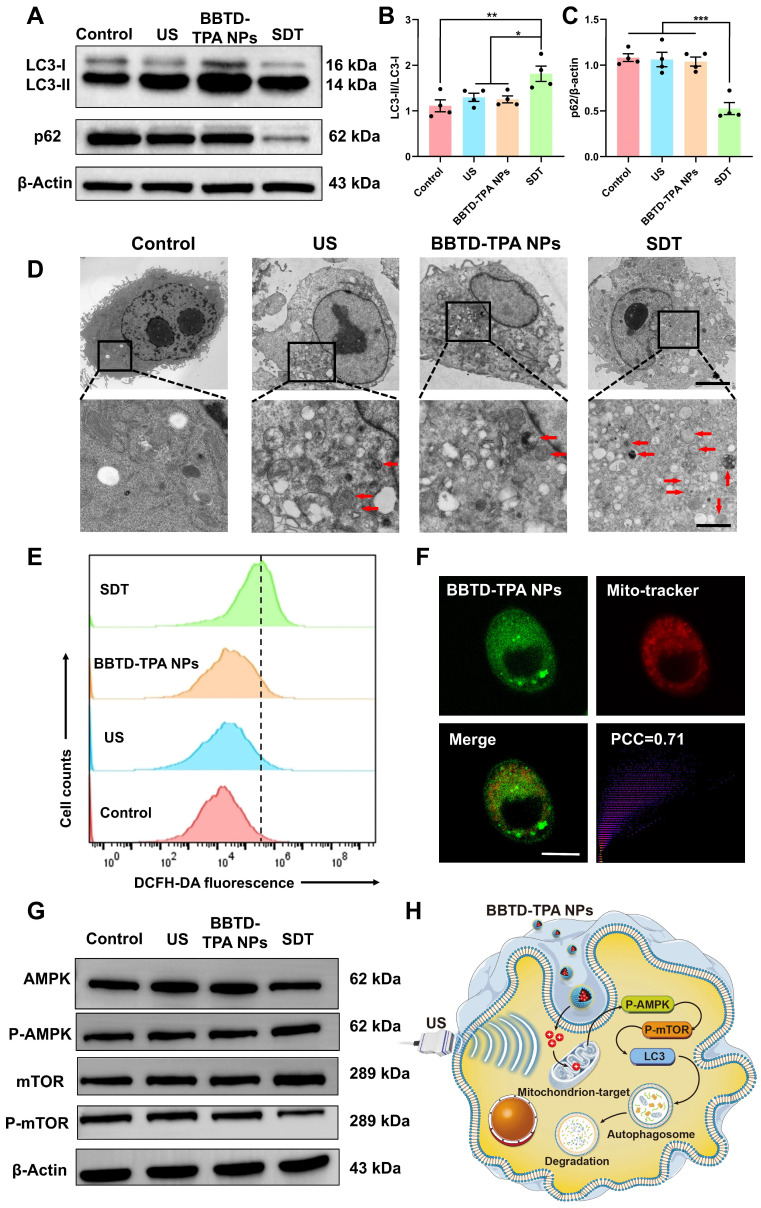
** BBTD-TPA** NPs-mediated SDT induced microglial autophagy through ROS-AMPK-mTOR signaling pathway. A-C) Western blot and quantitative analysis of LC3-II/I and p62 levels after different treatments. ^*^P < 0.05, ^**^P < 0.01, and ^***^P < 0.001. D) Representative TEM images of BV2 cells. Scale bar = 5 μm (above), 1 μm (below). Red arrows indicate autolysosomes. E) Flow cytometric analysis of DCFH-DA fluorescence intensity to detect ROS generation. F) Mitochondrial colocalization images of **BBTD-TPA** NPs in BV2 cells. Scale bar = 100 μm. G) Western blot assay of AMPK, P-AMPK, mTOR and P-mTOR in BV2 cells. Full uncropped blots are presented in **Figure S20**. H) Schematic illustration of mitochondria-targeted SDT-induced autophagy in BV2 cells.

Previous studies have demonstrated that SDT can induce apoptosis and autophagy, which is correlated with the dose of ROS produced. In general, low doses of ROS induce cellular autophagy, while excessive ROS induce apoptosis 40,41. Therefore, it is essential to regulate ROS production within an appropriate range to facilitate autophagy-enhanced SDT. To explore the optimal concentration of **BBTD-TPA** NPs for sonodynamic modulation, we examined the effect of different concentrations (5-80 μM) of **BBTD-TPA** NPs in BV2 cell viability using CCK-8 assay. The results indicated that concentrations below 40 μM did not result in obvious cell death in the presence of US irradiation, while concentrations above 60 μM led to a significant decrease in cell viability (**Figure S14**). To further investigate the effects of different concentrations of **BBTD-TPA** NPs on the autophagy levels of BV2 cells, microtubule-associated protein light chain 3 (LC3) immunofluorescence staining was performed. The findings indicated that 40 μM of **BBTD-TPA** NPs combined with ultrasound (1.0 MHz, 1.0 W cm^-2^, 5 min) could elicit the most pronounced autophagy induction in BV2 cells (**Figure S15**). Therefore, we adopted a concentration of 40 μM for subsequent studies to explore the effect of **BBTD-TPA** NPs-mediated SDT on cellular autophagy. LC3 is an autophagy-associated protein, and the transition from LC3-I to LC3-II, represents autophagosome formation 42,43. Following autophagosome formation, it fuses with lysosomes to form autolysosomes, where the autophagy substrate p62 is degraded 44. The results of the western blot indicated that the SDT group upregulated the ratio of LC3-II/I and decreased the level of the autophagy substrate p62, demonstrating an upregulation in autophagy (**Figure 3A-C**). Moreover, the TEM images revealed an increase in the number of autolysosomes in SDT-treated BV2 cells (**Figure 3D**). These findings suggested that **BBTD-TPA** NPs (40 μM)-mediated SDT might induce the upregulation of microglial autophagy.

Additionally, we quantified the generation of ROS using flow cytometry. The results indicated that the SDT group exhibited a mild increase in intracellular ROS levels (**Figure 3E** and **Figure S16**), which was consistent with the results of DCFH-DA fluorescence staining (**Figure S17**). Mitochondria are vital organelles for cellular ROS production and intricately linked to the occurrence of cellular autophagy 45,46. To further investigate the mitochondrial targeting of **BBTD-TPA** NPs, we co-incubated BV2 cells with the commercially available mitochondrial green probe (Mito-Tracker Green), **BBTD-TPA** NPs. The co-localization images demonstrated that **BBTD-TPA** NPs were predominantly localized to mitochondria, with a Pearson correlation coefficient (PCC) of 0.71 (**Figure 3F**). Moreover, JC-1 staining was employed to evaluate the effect of **BBTD-TPA** NPs-mediated SDT on mitochondrial membrane potential (MMP). As depicted in **Figure S18**, BV2 cells in the control, US, and **BBTD-TPA** NPs group exhibited distinct red fluorescence. In contrast, the SDT group showed strong green fluorescence, indicating that **BBTD-TPA** NPs-mediated SDT could target mitochondria and affect MMP. To further investigate the molecular mechanism by which SDT promotes cellular autophagy, we analyzed the levels of 5' AMP-activated protein kinase (AMPK), phosphorylated AMPK (P-AMPK), mammalian target of rapamycin (mTOR), and phosphorylated mTOR (p-mTOR) in BV2 cells after different treatments. We found that SDT treatment increased the phosphorylation of AMPK and decreased the level of p-mTOR (**Figure 3G** and **Figure S19**). AMPK is an energy-sensitive enzyme that senses cellular energy levels and regulates cellular metabolism 47. During energy stress or mitochondrial injury, AMPK is phosphorylated and activated, which further inhibits mTOR and induces autophagy 48. These results demonstrated that **BBTD-TPA** NPs-mediated SDT may target mitochondria and promote microglial autophagy through the ROS-AMPK-mTOR pathway, thus modulating neuroinflammation (**Figure 3H**).

### NIR-II fluorescence imaging and the distribution of BBTD-TPA NPs *in vivo*

**Figure 4:**
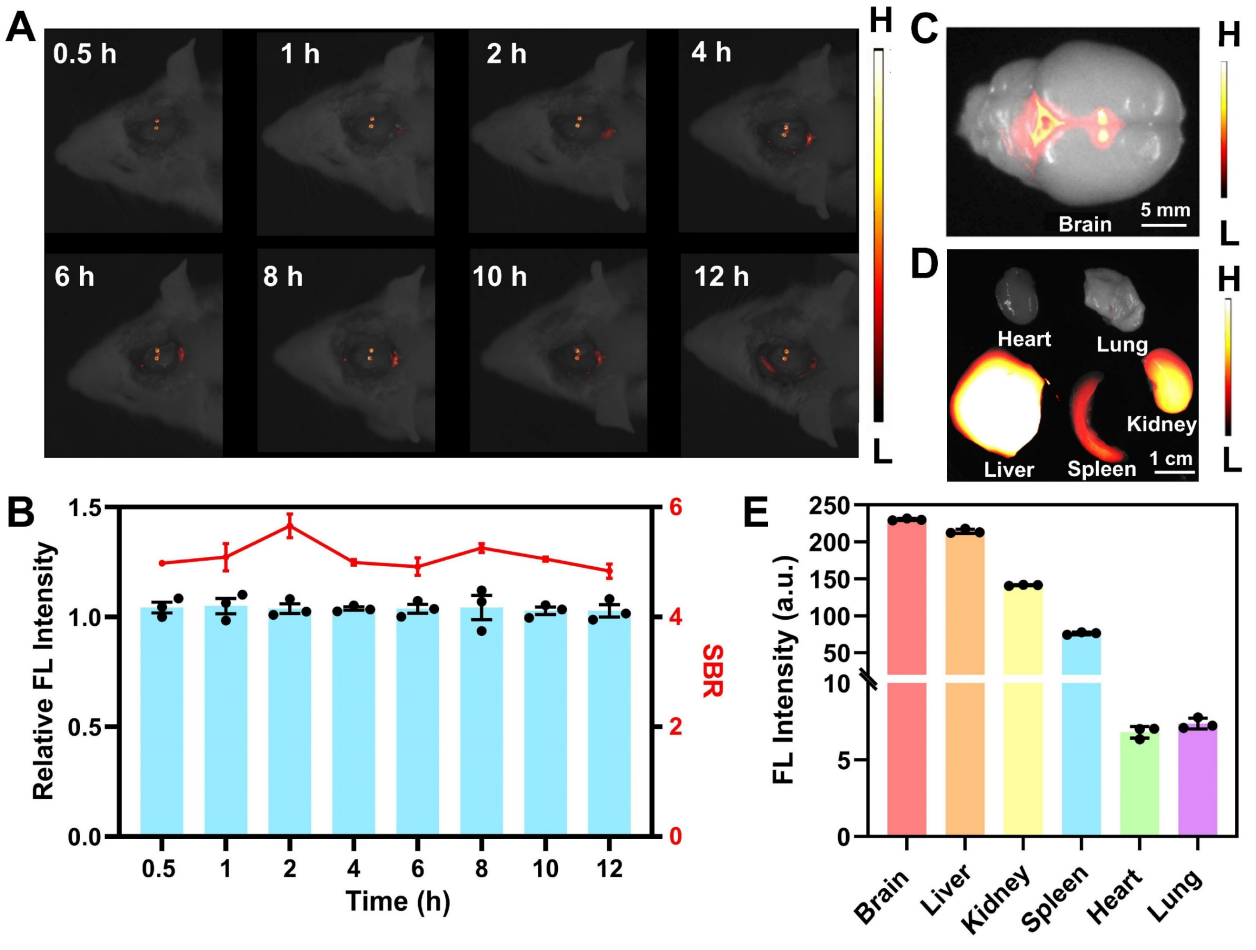
NIR-II fluorescence imaging of **BBTD-TPA** NPs *in vivo*. A) *In vivo* fluorescence imaging results with the injection of **BBTD-TPA** NPs in the PVN. B) Analysis of relative fluorescence intensity and SBR at different time points. *Ex vivo* fluorescence images of the C) brain and D) other major organs after injection of **BBTD-TPA** NPs for 12 h. Scale bar = 5 mm (above), 1 cm (below). E) Quantitative analysis of NIR-II fluorescence intensity of *ex vivo* organs.

In preparation for subsequent *in vivo* imaging, hemolysis assays were conducted to evaluate the biosafety of **BBTD-TPA** NPs. The red blood cells were incubated with varying concentrations of **BBTD-TPA** NPs, namely 25, 50, 100, 200, and 400 µM. The corresponding hemolysis rates were all less than 2.5% (**Figure S21**). The results indicated that **BBTD-TPA** NPs exhibited high hemocompatibility. Utilizing the properties of NIR-II fluorescence imaging, we performed local imaging of the PVN by **BBTD-TPA** NPs to assess their metabolism and distribution *in vivo*. **BBTD-TPA** NPs (400 μM, 5 μL) were injected into the bilateral PVN, and *in vivo* real-time imaging was performed over the following 12 h. The result indicated that **BBTD-TPA** NPs concentrated in the PVN and remained there for up to 12 h (**Figure 4A**). Quantitative fluorescence analysis demonstrated that the fluorescence intensity and signal-to-background ratio (SBR) of the PVN remained stable throughout the 12-hour period (**Figure 4B**). At 12 h after injection, the brain and other vital organs were imaged *ex vivo*. The results showed that **BBTD-TPA** NPs were mainly enriched in the PVN region and the third ventricle (**Figure 4C**). In addition, the liver, spleen, and kidney also showed high fluorescence signals, suggesting that **BBTD-TPA** NPs may be metabolized *in vivo* through the liver and kidney (**Figure 4D, E**).

### SDT inhibited MI-induced sympathetic hyperactivity and reversed high sympathetic tone

**Figure 5:**
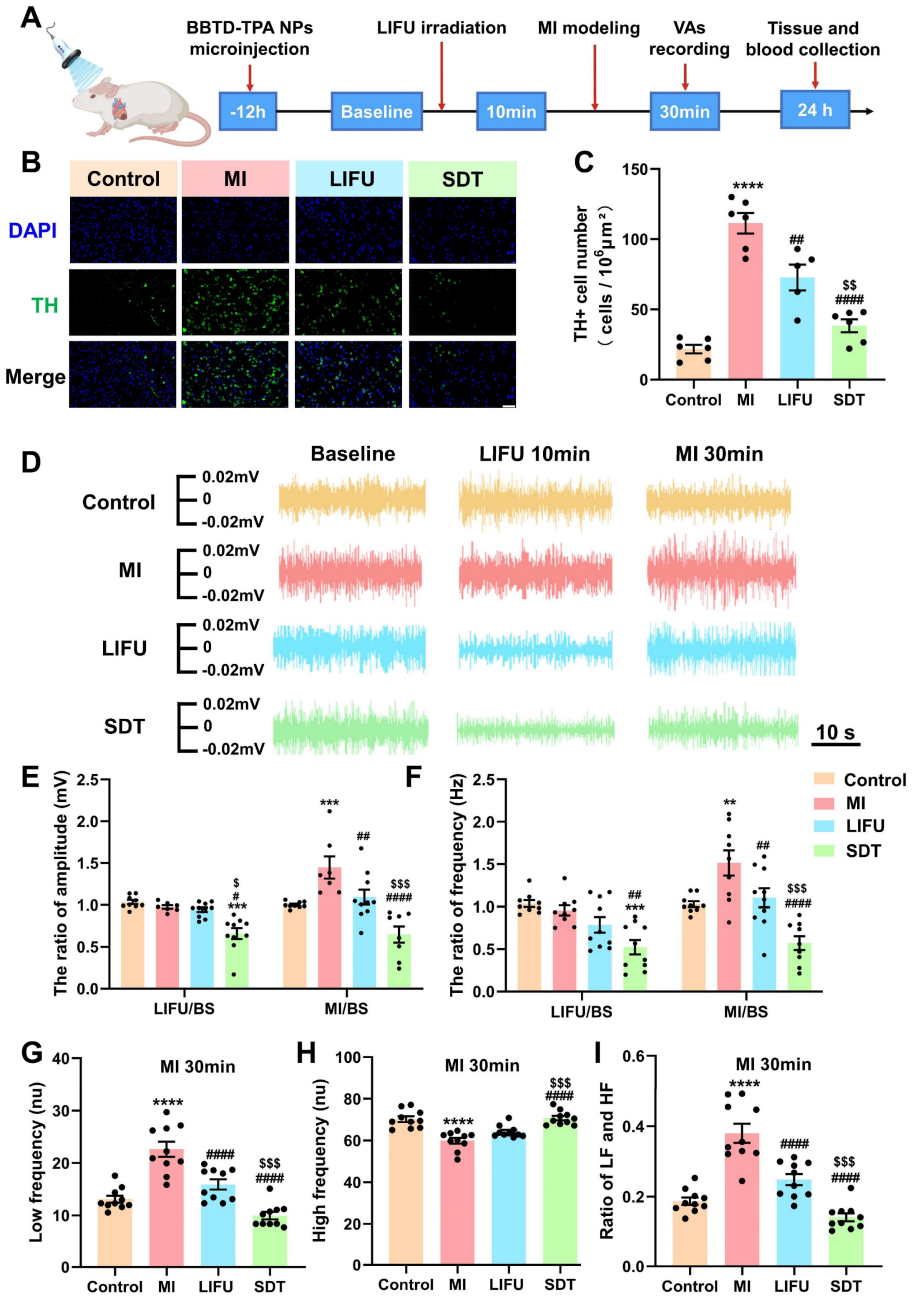
** BBTD-TPA** NPs-mediated SDT attenuated central sympathetic neural activity and LSG neural activity (1.0 MHz, 2.0 W cm^-2^). A) Flowchart of *in vivo* experiments. B) Representation of TH immunofluorescence staining in the PVN in the four groups. Scale bar = 100 μm. C) Quantitative analysis of TH+ cell number (n = 6 per group). D) Representative LSG neural activity recordings of these four groups at different time points. E, F) Statistical analysis of the amplitude and frequency of LSG in the four groups (n = 10 per group). G-I) HRV analysis in the four groups at MI 30 min (n = 10 per group). ^**^P < 0.01, ^***^P < 0.001, and ^****^P < 0.0001 versus the control group; *^#^P < 0.05*, *^##^P < 0.01*, and *^####^P < 0.0001* versus the MI group; *^$^P < 0.05*, *^$$^P < 0.01*, and *^$$$^P < 0.001* versus the LIFU group.

The flowchart of the *in vivo* experiment is outlined in **Figure 5A** and **Figure S22**. Forty rats were randomized equally to the control group, MI group, LIFU group and SDT group. The sonosensitizer **BBTD-TPA** NPs (400 μM, 5μL) were microinjected into the PVN of rats in the SDT group, and saline was injected in the remaining groups. Among them, the SDT and LIFU groups were treated with ultrasound, and the MI and control groups were given sham ultrasound stimulation. In addition, all groups except the control group underwent modeling of acute MI. LSG neural activity recordings, effective refractory period (ERP) measurements, and arrhythmia evaluation were performed in all groups of rats. At the termination of the experiment, the rat brain and heart tissues were subjected to histological examination, and major organs and blood samples were extracted for biosafety assessment.

Tyrosine hydroxylase (TH) immunofluorescence staining in the PVN indicates the activity of central sympathetic neurons 49. **Figure 5B** shows representative images of TH staining of PVN tissue in the four groups. Compared with the control group, MI caused excessive activation of TH+ sympathetic neurons, while LIFU and SDT suppressed this activation. Notably, SDT had a significant inhibitory effect compared to that of LIFU (**Figure 5C**). To assess peripheral sympathetic activity, we recorded the amplitude and frequency of LSG discharges over different time periods (**Figure 5D**). Quantitative statistical results showed that LIFU and SDT could inhibit LSG neural activity after ultrasonic intervention and after MI modeling. Moreover, SDT decreased LSG neural activity more significantly than LIFU (**Figure 5E, F**). As a key indicator of systemic autonomic tone, HRV includes LF, HF and the ratio of LF/HF. LF and HF were used to represent sympathetic and parasympathetic tone, respectively 37,50. The results demonstrated that MI resulted in a significant increase in LF and LF/HF ratio and a decrease in HF compared to the control group. However, LIFU and SDT reversed these alterations, with SDT demonstrating a more pronounced effect than LIFU (**Figure 5G-I** and **Figure S23**).

### SDT attenuated post-MI nervous, myocardial and systemic inflammation

**Figure 6:**
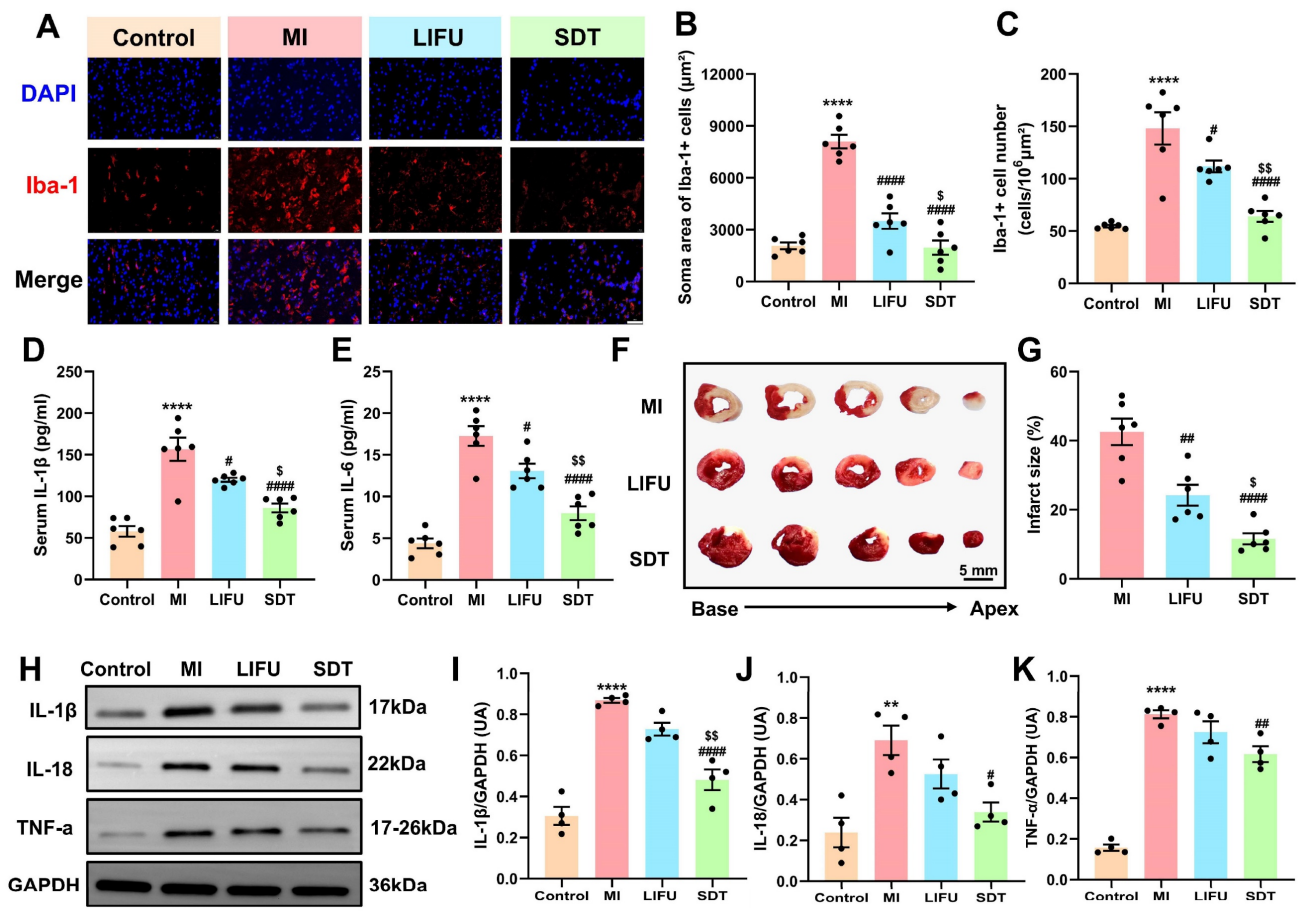
**BBTD-TPA** NPs-mediated SDT ameliorate post-MI nervous, systemic, and myocardial inflammatory responses. A) Representative images of Iba-1 immunofluorescence staining in the PVN in various groups. Scale bar = 100 μm. B, C) Quantitative analysis of the Iba-1+ cell number and soma area. D, E) Serum levels of pro-inflammatory cytokines IL-1β and IL-6 (n = 6 per group). F) Typical images of TTC staining in various group. The red area represented a region with a normal blood supply, while the white area represented the infarct size. Scale bar = 5 mm. G) Quantitative analysis of myocardial infarct size (n = 6 per group). H-K) Western blot and quantitative analysis of IL-1β, IL-18, and TNF-α in peri-infarct myocardium (n = 4 per group). Full uncropped blots are presented in **Figure S26**. ^**^P < 0.01, and^ ****^P < 0.0001 versus the control group; *^#^P < 0.05*, *^##^P < 0.01*, and *^####^P < 0.0001* versus the MI group; *^$^P < 0.05*, and *^$$^P < 0.01* versus the LIFU group.

As brain-resident immune cells, microglia play a crucial role in monitoring and regulating at the synaptic and neuronal levels 6. Previous studies have indicated that microglial activation are involved in neuroinflammation in cardiovascular diseases such as hypertension, MI, and ischemia‒reperfusion injury 6,51,52. To explore the effect of SDT on neuroinflammation, ionized calcium binding adaptor molecule-1 (Iba-1) immunofluorescence staining was employed to label microglia within the PVN. The representative images of immunofluorescence staining are shown in **Figure 6A**. Compared to the control group, the MI group exhibited significantly activated microglia, and this activation effect was attenuated by LIFU and SDT. Notably, SDT decreased microglial activation and neuroinflammation more significantly than LIFU alone (soma area: 1962.61 ± 408.84 vs. 3499.23 ± 449.09, *P < 0.05* vs. the LIFU group; cell number: 64.08 ± 5.21 vs. 111.80 ± 5.60, *P < 0.01* vs. the LIFU group) (**Figure 6B, C**). To evaluate the ROS generation capacity of **BBTD-TPA** NPs *in vivo*, PVN tissues were stained with DHE (**Figure S24**). Quantitative analysis indicated a significant increase in the area of DHE+ cell area in the SDT group. However, there was no significant difference in the DHE+ cell number compared to the other groups (*P > 0.05*). To further validate the effect of SDT on autophagy levels *in vivo*, we performed LC3 immunofluorescence staining of PVN regions. The results demonstrated that LC3+ cell number in the SDT group was significantly elevated compared to the other three groups, indicating that SDT could upregulate the level of cellular autophagy within the PVN (**Figure S25**).

Previous studies have demonstrated that there is an enhanced inflammatory response characterized by elevated serum cytokines and increased recruitment and retention of inflammatory cells in the peri-infarct zone after MI, which exacerbates the cardiac electrophysiological remodeling and the occurrence of arrhythmias 53-55. To investigate the effect of SDT on post-MI systemic inflammation, we employed enzyme-linked immunosorbent assay (ELISA) to quantify serum levels of the pro-inflammatory cytokines including interleukin-1β (IL-1β) and interleukin-6 (IL-6). The results revealed that MI significantly upregulated serum IL-1β and IL-6 levels in comparison to the control group (**Figure 6D, E**). Conversely, LIFU and SDT demonstrated the capacity to downregulate this alteration, suggesting that LIFU and SDT modulation of PVN might suppress systemic inflammation. Additionally, we employed triphenyltetrazolium chloride (TTC) staining to assess the myocardial infarction size (**Figure 6F**). The results demonstrated that LIFU and SDT significantly reduced infarct size compared with the MI group, thereby exerting myocardial protective effect (**Figure 6G**). Notably, the protective effect of the SDT group was superior to that of LIFU intervention alone. Furthermore, western blot was employed to detect the relative levels of cytokines, including IL-1β, interleukin-18 (IL-18), and tumor necrosis factor-α (TNF-α) of the peri-infarct myocardium (**Figure 6H**). The findings demonstrated that MI significantly upregulated these cytokines, whereas SDT exhibited a suppressive effect on the expression these cytokines (**Figure 6I-K**). Compared to the LIFU alone, **BBTD-TPA** NPs combined with LIFU appeared to enhance the inhibitory impact on the myocardial inflammatory response. The current results suggested that **BBTD-TPA** NPs-mediated SDT might ameliorate post-MI neuroinflammation, systemic inflammation, and myocardial inflammation, which might be crucial in preventing MI-induced VAs.

### SDT reduced the occurrence of post-MI VA episodes

**Figure 7:**
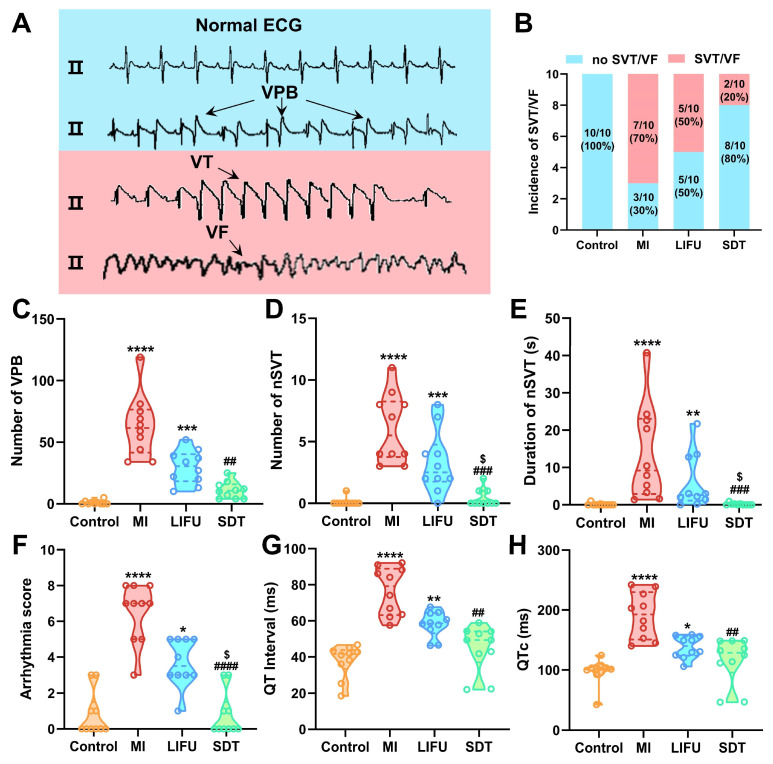
The occurrence of VAs and ventricular electrophysiological properties in different groups. A) Typical image of normal ECG, VPB, VT and VF. B) The incidence of SVT/VF, C-E) the incidence and duration of VPB and nSVT, F) arrhythmia score, G) QT intervals and H) corrected QT interval (QTc) in the four groups. Data are expressed as median with interquartile range (n = 10 per group). ^*^P < 0.05, ^**^P < 0.01, ^***^P < 0.001, and ^****^P < 0.0001 versus the control group; *^##^P < 0.01*, *^###^P < 0.001*, and *^####^P < 0.0001* versus the MI group; *^$^P < 0.05* versus the LIFU group.

VAs are known as the leading cause of sudden cardiac death in patients with MI, mainly including VPB, nSVT, SVT, and VF 36,56. Typical ECG of VAs induced by MI is shown in **Figure 7A**. It is well established that SVT and VF are the most serious and lethal form of arrhythmia. In the current study, the incidence of SVT/VF in the MI group reached 70%, which was reduced by LIFU and SDT intervention (**Figure 7B**). Furthermore, the incidence of SVT/VF appeared to be lower in the SDT group. In comparison to the control group, MI significantly increased the number of VPBs and nSVTs, the duration of nSVTs and the arrhythmia score. LIFU intervention had a tend to reduce the occurrence of post-MI VAs and decrease the arrhythmia score. Satisfactorily, SDT enhanced the effect of LIFU and reduced the MI-induced VAs and arrhythmia score more significantly (number of VPB: 10.50 [4.00, 15.75] vs. 61.50 [41.50, 76.50], *P < 0.01* vs. the MI group; number of nSVT: 0.00 [0.00, 1.00] vs. 5.50 [3.75, 8.25], *P < 0.001* vs. the MI group; duration of nSVT: 0.00 [0.00, 0.22] vs. 9.16 [2.90, 23.04], *P < 0.001* vs. the MI group; arrhythmia score: 0.00 [0.00, 1.50] vs. 7.00 [5.00, 8.00], *P < 0.0001* vs. the MI group) (**Figure 7C-F**). Furthermore, we measured the QT interval and corrected QT interval (QTc) on the post-MI ECG. The results showed that SDT interventions reversed the MI-induced prolongation of QT interval and QTc (**Figure 7G, H**). In addition, as an essential electrophysiological feature of the ventricle, ERP has important predictive value for arrhythmias. Consequently, we examined the ERP of the left ventricular apex, middle, and bottom of the myocardium. After ultrasonic intervention, there was no significant variance in ERP in the four groups. After MI modeling, the ERP was significantly shortened in the MI group compared to the control group, while SDT and LIFU prolonged the ERP to a certain extent and offset the adverse effects of MI on ventricular ERP (**Figure S27A-C**).

### Biocompatibility assessment of BBTD-TPA NPs and SDT *in vivo*

To evaluate the thermal effect of LIFU and SDT, we used a thermal camera to measure the temperature of the PVN before and after ultrasound treatment, and the results showed no significant change in temperature (**Figure S28A-C**). After treatment, H&E staining of the heart (distal infarction area), lung, liver, spleen and kidney from the rats in the four groups was conducted to assess the biosafety of LIFU and **BBTD-TPA** NPs-mediated SDT. No apparent pathological injury was observed in these tissues (**Figure S29**). Moreover, the blood tests showed no notable deviations from normal levels in blood cell count, liver and kidney functions after the LIFU and SDT intervention (**Figure S30** and **Figure S31**), suggesting the high biocompatibility and biosafety of **BBTD-TPA** NPs and SDT.

## Conclusion

In summary, we constructed an autophagy-enhanced nanosonosensitizer **BBTD-TPA** NPs for SDT with long-wavelength emission, mitochondria targeting, and high biocompatibility. To the best of our knowledge, this work is the inaugural attempt to apply SDT for neuromodulation and arrhythmia prevention. We demonstrated that **BBTD-TPA** NPs combined with LIFU might promote the microglial autophagy through the ROS-AMPK-mTOR pathway. Moreover, **BBTD-TPA** NPs-mediated SDT could inhibit the sympathetic hyperactivation and ameliorate inflammatory response after MI, thus preventing the occurrence of MI-induced VAs. Therefore, SDT could be highlighted as a noninvasive, high spatial resolution, deep penetration, efficient and biosafe strategy to prevent the occurrence of post-MI VAs.

## Supplementary Material

Supplementary methods, figures and information.

## Abbreviations

AMPK: 5' AMP-activated protein kinase
BBTD: benzo[1,2-c:4,5c′]bis[1,2,5]thiadiazole
BS: baseline
CCK-8: cell counting kit-8
DCFH-DA: dichlorofluorescein diacetate
DHE: dihydroethidium
DLS: dynamic light scattering
DPBF: 1,3-Diphenylisobenzofurandiphenylisobenzofuran
ECG: electrocardiogram
ELISA: enzyme-linked immunosorbent assay
ERP: effective refractory period
HF: high frequency
HRV: heart rate variability
Iba-1: ionized calcium binding adaptor molecule-1
IL-18: interleukin-18
IL-1β: interleukin-1β
IL-6: interleukin-6
LAD: left anterior descending artery
LC3: light chain 3
LED: light-emitting diode
LF: low frequency
LIFU: low-intensity focused ultrasound
LSG: left stellate ganglion
MI: myocardial infarction
MMP: mitochondrial membrane potential
mTOR: mammalian target of rapamycin
NIR: near-infrared light
NPs: nanoparticles
nSVT: non-sustained ventricular tachycardia
P-AMPK: phosphorylated 5' AMP-activated protein kinase
PDT: photodynamic therapy
P-mTOR: phosphorylated mammalian target of rapamycin
PTT: photothermal therapy
PVN: paraventricular nucleus
QTc: corrected QT interval
ROS: reactive oxygen species
SBR: signal-to-background ratio
SDT: sonodynamic therapy
SNA: sympathetic nervous activity
SVT: sustained ventricular tachycardia
TEM: transmission electron microscopy
TH: tyrosine hydroxylase
TNF-α: tumor necrosis factor-α
TPA: triphenylamine
TTC: triphenyltetrazolium chloride
US: ultrasound
VA: ventricular arrhythmia
VF: ventricular fibrillation
VPB: premature ventricular beat
VT: ventricular tachycardia

## References

[1] John RM, Tedrow UB, Koplan BA, Albert CM, Epstein LM, Sweeney MO (2012). Ventricular arrhythmias and sudden cardiac death. Lancet.

[2] Bunch TJ, Hohnloser SH, Gersh BJ (2007). Mechanisms of sudden cardiac death in myocardial infarction survivors. Circulation.

[3] Priori SG, Blomstrom-Lundqvist C, Mazzanti A, Blom N, Borggrefe M, Camm J (2015). 2015 ESC Guidelines for the management of patients with ventricular arrhythmias and the prevention of sudden cardiac death: the task force for the management of patients with ventricular arrhythmias and the prevention of sudden cardiac death of the european society of cardiology (ESC). Eur Heart J.

[4] Herring N, Kalla M, Paterson DJ (2019). The autonomic nervous system and cardiac arrhythmias: current concepts and emerging therapies. Nat Rev Cardiol.

[5] Kabir RA, Doytchinova A, Liu X, Adams D, Straka S, Chen LS (2017). Crescendo skin sympathetic nerve activity and ventricular arrhythmia. J Am Coll Cardiol.

[6] Wang M, Pan W, Xu Y, Zhang J, Wan J, Jiang H (2022). Microglia-mediated neuroinflammation: a potential target for the treatment of cardiovascular diseases. J Inflamm Res.

[7] Felder RB, Francis J, Zhang ZH, Wei SG, Weiss RM, Johnson AK (2003). Heart failure and the brain: new perspectives. Am J Physiol Regul Integr Comp Physiol.

[8] Santisteban MM, Ahmari N, Carvajal JM, Zingler MB, Qi Y, Kim S (2015). Involvement of bone marrow cells and neuroinflammation in hypertension. Circ Res.

[9] Rana I, Stebbing M, Kompa A, Kelly DJ, Krum H, Badoer E (2010). Microglia activation in the hypothalamic PVN following myocardial infarction. Brain Res.

[10] Schwartz PJ, Ackerman MJ (2022). Cardiac sympathetic denervation in the prevention of genetically mediated life-threatening ventricular arrhythmias. Eur Heart J.

[11] Schwartz PJ, Stone HL (1980). Left stellectomy in the prevention of ventricular fibrillation caused by acute myocardial ischemia in conscious dogs with anterior myocardial infarction. Circulation.

[12] Bourke T, Vaseghi M, Michowitz Y, Sankhla V, Shah M, Swapna N (2010). Neuraxial modulation for refractory ventricular arrhythmias: value of thoracic epidural anesthesia and surgical left cardiac sympathetic denervation. Circulation.

[13] Wang S, Li B, Li X, Wu L, Zhu T, Zhao D (2020). Low-intensity ultrasound modulation may prevent myocardial infarction-induced sympathetic neural activation and ventricular arrhythmia. J Cardiovasc Pharmacol.

[14] Ye T, Lai Y, Wang Z, Zhang X, Meng G, Zhou L (2019). Precise modulation of gold nanorods for protecting against malignant ventricular arrhythmias via near-infrared neuromodulation. Adv Funct Mater.

[15] Wang S, Wu L, Li X, Li B, Zhai Y, Zhao D (2019). Light-emitting diode therapy protects against ventricular arrhythmias by neuro-immune modulation in myocardial ischemia and reperfusion rat model. J Neuroinflammation.

[16] Xiang C, Cheng Y, Yu X, Mao T, Luo H, Hu H (2024). Low-intensity focused ultrasound modulation of the paraventricular nucleus to prevent myocardial infarction-induced ventricular arrhythmia. Heart Rhythm.

[17] Son S, Kim JH, Wang X, Zhang C, Yoon SA, Shin J (2020). Multifunctional sonosensitizers in sonodynamic cancer therapy. Chem Soc Rev.

[18] Zhao P, Deng Y, Xiang G, Liu Y (2021). Nanoparticle-assisted sonosensitizers and their biomedical applications. Int J Nanomedicine.

[19] Alves F, Pavarina AC, Mima EGO, McHale AP, Callan JF (2018). Antimicrobial sonodynamic and photodynamic therapies against Candida albicans. Biofouling.

[20] Geng C, Zhang Y, Hidru TH, Zhi L, Tao M, Zou L (2018). Sonodynamic therapy: a potential treatment for atherosclerosis. Life Sci.

[21] Wang X, Wu M, Li H, Jiang J, Zhou S, Chen W (2022). Enhancing penetration ability of semiconducting polymer nanoparticles for sonodynamic therapy of large solid tumor. Adv Sci.

[22] Wu Q, Zhang J, Pan X, Huang Z, Zhang H, Guo J (2023). Vacancy augmented piezo-sonosensitizer for cancer therapy. Adv Sci.

[23] Zhang Y, Zhang X, Yang H, Yu L, Xu Y, Sharma A (2021). Advanced biotechnology-assisted precise sonodynamic therapy. Chem Soc Rev.

[24] An J, Hu YG, Cheng K, Li C, Hou XL, Wang GL (2020). ROS-augmented and tumor-microenvironment responsive biodegradable nanoplatform for enhancing chemo-sonodynamic therapy. Biomaterials.

[25] You DG, Deepagan VG, Um W, Jeon S, Son S, Chang H (2016). ROS-generating TiO2 nanoparticles for non-invasive sonodynamic therapy of cancer. Sci Rep.

[26] Yang Y, Wang J, Guo S, Pourteymour S, Xu Q, Gong J (2020). Non-lethal sonodynamic therapy facilitates the M1-to-M2 transition in advanced atherosclerotic plaques via activating the ROS-AMPK-mTORC1-autophagy pathway. Redox Biol.

[27] Berglund R, Guerreiro-Cacais AO, Adzemovic MZ, Zeitelhofer M, Lund H, Ewing E (2020). Microglial autophagy-associated phagocytosis is essential for recovery from neuroinflammation. Sci Immunol.

[28] Su P, Zhang J, Wang D, Zhao F, Cao Z, Aschner M (2016). The role of autophagy in modulation of neuroinflammation in microglia. Neuroscience.

[29] Xing X, Zhao S, Xu T, Huang L, Zhang Y, Lan M (2021). Advances and perspectives in organic sonosensitizers for sonodynamic therapy. Coord Chem Rev.

[30] Li D, Yang Y, Li D, Pan J, Chu C, Liu G (2021). Organic sonosensitizers for sonodynamic therapy: from small molecules and nanoparticles toward clinical development. Small.

[31] Nguyen Cao TG, Truong Hoang Q, Kang JH, Kang SJ, Ravichandran V, Rhee WJ (2023). Bioreducible exosomes encapsulating glycolysis inhibitors potentiate mitochondria-targeted sonodynamic cancer therapy via cancer-targeted drug release and cellular energy depletion. Biomaterials.

[32] Yang Z, Yuan M, Liu B, Zhang W, Maleki A, Guo B (2022). Conferring BiVO4 nanorods with pxygen vacancies to realize enhanced sonodynamic cancer therapy. Angew Chem Int Ed Engl.

[33] Chen T, Zeng W, Liu Y, Yu M, Huang C, Shi Z (2022). Cu-doped polypyrrole with multi-catalytic activities for sono-enhanced nanocatalytic tumor therapy. Small.

[34] Wang J, Zhao Z, Liu Y, Cao X, Li F, Ran H (2022). 'Mito-bomb': a novel mitochondria-targeting nanosystem for ferroptosis-boosted sonodynamic antitumor therapy. Drug Deliv.

[35] Li J, Dong Y, Wei R, Jiang G, Yao C, Lv M (2022). Stable, bright, and long-fluorescence-lifetime dyes for deep-near-infrared bioimaging. J Am Chem Soc.

[36] Yu L, Zhou L, Cao G, Po SS, Huang B, Zhou X (2017). Optogenetic modulation of cardiac sympathetic nerve activity to prevent ventricular arrhythmias. J Am Coll Cardiol.

[37] Lai Y, Zhou X, Guo F, Jin X, Meng G, Zhou L (2022). Non-invasive transcutaneous vagal nerve stimulation improves myocardial performance in doxorubicin-induced cardiotoxicity. Cardiovasc Res.

[38] Torchilin VP (2005). Recent advances with liposomes as pharmaceutical carriers. Nat Rev Drug Discov.

[39] Hu R, Chen B, Wang Z, Qin A, Zhao Z, Lou X (2019). Intriguing “chameleon” fluorescent bioprobes for the visualization of lipid droplet-lysosome interplay. Biomaterials.

[40] Chen F, Xue Q, He N, Zhang X, Li S, Zhao C (2023). The association and application of sonodynamic therapy and autophagy in diseases. Life Sci.

[41] Su X, Wang P, Yang S, Zhang K, Liu Q, Wang X (2015). Sonodynamic therapy induces the interplay between apoptosis and autophagy in K562 cells through ROS. Int J Biochem Cell Biol.

[42] Kirkin V, Rogov VV (2019). A diversity of selective autophagy receptors determines the specificity of the autophagy pathway. Mol Cell.

[43] Yuan J, Liu H, Zhang H, Wang T, Zheng Q, Li Z (2022). Controlled activation of TRPV1 channels on microglia to boost their autophagy for clearance of alpha-synuclein and enhance therapy of Parkinson's disease. Adv Mater.

[44] Mizushima N, Levine B, Cuervo AM, Klionsky DJ (2008). Autophagy fights disease through cellular self-digestion. Nature.

[45] Willems Peter HGM, Rossignol R, Dieteren Cindy EJ, Murphy Michael P, Koopman Werner JH (2015). Redox homeostasis and mitochondrial dynamics. Cell Metab.

[46] Li A, Gao M, Liu B, Qin Y, chen L, Liu H (2022). Mitochondrial autophagy: molecular mechanisms and implications for cardiovascular disease. Cell Death Dis.

[47] Herzig S, Shaw RJ (2017). AMPK: guardian of metabolism and mitochondrial homeostasis. Nat Rev Mol Cell Biol.

[48] Poels J, Spasić MR, Callaerts P, Norga KK (2009). Expanding roles for AMP-activated protein kinase in neuronal survival and autophagy. BioEssays.

[49] Shi Y-C, Lau J, Lin Z, Zhang H, Zhai L, Sperk G (2013). Arcuate NPY controls sympathetic putput and BAT function via a relay of tyrosine hydroxylase neurons in the PVN. Cell Metab.

[50] Khan AA, Lip GYH, Shantsila A (2019). Heart rate variability in atrial fibrillation: The balance between sympathetic and parasympathetic nervous system. Eur J Clin Invest.

[51] Shen XZ, Li Y, Li L, Shah KH, Bernstein KE, Lyden P (2015). Microglia participate in neurogenic regulation of hypertension. Hypertension.

[52] Menz MD, Oralkan O, Khuri-Yakub PT, Baccus SA (2013). Precise neural stimulation in the retina using focused ultrasound. J Neurosci.

[53] De Jesus NM, Wang L, Herren AW, Wang J, Shenasa F, Bers DM (2015). Atherosclerosis exacerbates arrhythmia following myocardial infarction: role of myocardial inflammation. Heart Rhythm.

[54] Ziegler KA, Ahles A, Wille T, Kerler J, Ramanujam D, Engelhardt S (2018). Local sympathetic denervation attenuates myocardial inflammation and improves cardiac function after myocardial infarction in mice. Cardiovas Res.

[55] Peretto G, Sala S, Rizzo S, Palmisano A, Esposito A, De Cobelli F (2020). Ventricular arrhythmias in myocarditis: characterization and relationships with myocardial inflammation. J Am Coll Cardiol.

[56] Wang S, Wu L, Zhai Y, Li X, Li B, Zhao D (2019). Noninvasive light emitting diode therapy: A novel approach for postinfarction ventricular arrhythmias and neuroimmune modulation. J Cardiovasc Electrophysiol.

