# Supplementary material for: Autophagy-enhanced nanosonosensitizer mediated sonodynamic therapy for post-myocardial infarction neuromodulation and arrhythmia prevention
Source: Theranostics. 2025 Jan 13;15(6):2201–14. doi: 10.7150/thno.103780 (PMC11840733; doi:10.7150/thno.103780)
Supplement: Supplementary file 1 — Supplementary methods, figures and information. [file thnov15p2201s1.pdf]

Supporting Information

Autophagy-enhanced nanosonosensitizer mediated sonodynamic therapy for post-myocardial infarction neuromodulation and arrhythmia prevention

Haoyuan Hu[1†]; Songyun Wang[1†]; Qian Li[2†]; Jiahui Zhao[1†]; Yida Pang[2]; Jiale Wang[1]; Huijun Wu[1]; Xinqi Wang[1]; Ye Cheng[1]; Mengran Yu[1]; Xinyue Yin[1]; Yan Zhang[1]; Lilei Yu[1]*; Yao Sun[2]*; Hong Jiang[1]*

1 Department of Cardiology, Renmin Hospital of Wuhan University; Cardiac Autonomic Nervous System Research Center of Wuhan University; Cardiovascular Research Institute, Wuhan University; Hubei Key Laboratory of Cardiology, Wuhan, China.
2 State Key Laboratory of Green Pesticide, International Joint Research Center for Intelligent Biosensor Technology and Health College of Chemistry, Central China Normal University.

† Haoyuan Hu, Songyun Wang, Qian Li, and Jiahui Zhao contributed equally to this work.

***Corresponding author:**
Hong Jiang
Department of Cardiology, Renmin Hospital of Wuhan University
Cardiac Autonomic Nervous System Research Center of Wuhan University
Cardiovascular Research Institute, Wuhan University
Hubei Key Laboratory of Cardiology
Wuhan 430061, China.
E-mail: hong-jiang@whu.edu.cn

Yao Sun
State Key Laboratory of Green Pesticide,
International Joint Research Center for Intelligent Biosensor Technology and Health
College of Chemistry, Central China Normal University
Wuhan 430079, China.
E-mail: sunyaogbasp@ccnu.edu.cn

Lilei Yu
Department of Cardiology, Renmin Hospital of Wuhan University
Cardiac Autonomic Nervous System Research Center of Wuhan University
Cardiovascular Research Institute, Wuhan University
Hubei Key Laboratory of Cardiology
Wuhan 430061, China.
E-mail: lileiyu@whu.edu.cn

Table of Contents

1. Supplementary Materials and Methods······S3

2. Supplementary Figures······S8

2.1 Images of PVN localization and ultrasound devices······S8

2.2 Characterization of **BBTD-TPA** NPs······S9

2.3 *In vitro* study of **BBTD-TPA** NPs······S18

2.4 *In vivo* study of **BBTD-TPA** NPs······S22

1. Supplementary Materials and Methods

Synthetic procedures and characterization of BBTD-TPA NPs

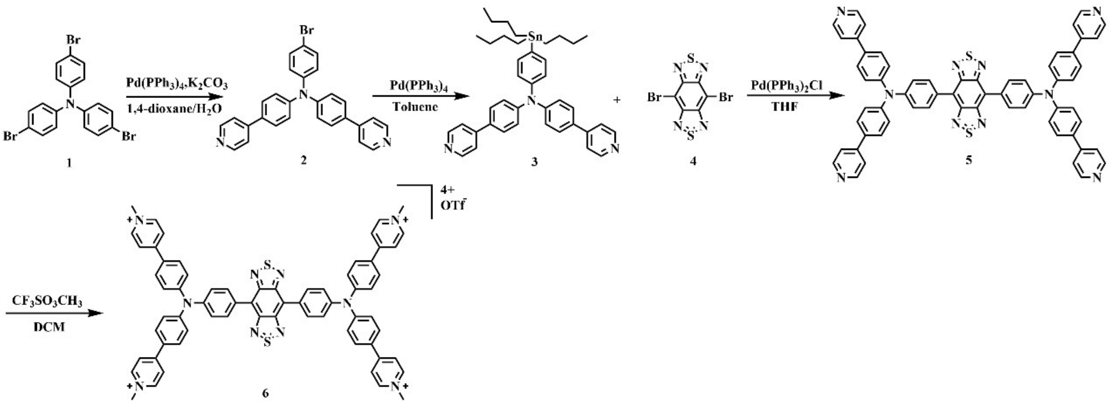

Synthesis of Compound **2**:

Under nitrogen protected conditions, the compound **1** (5.0 g, 10.3 mmol, 1.0 equiv.), 4-pyridyl boric acid (2.5 g, 20.6 mmol, 2 equiv.) and tetrakis(triphenylphosphine) palladium (0) (594 mg, 0.52 mmol, 0.05 equiv.) were dissolved in 1, 4-dioxane (180 mL), followed by K_2CO_3 aqueous solution (0.5 M, 50 mL). The reaction mixture was heated in an oil bath at 75°C for 12 h. After the reaction is complete, the reaction liquid is cooled to room temperature and extracted with methylene chloride and water. The organic phase was collected and dried with anhydrous sodium sulfate. The crude product was purified by silica gel column (EA: DCM = 1:1, V/V). yellow solid product **2** was obtained: 1.5 g, with a yield of 30%. ^{1}H NMR (400 MHz, $CDCl_3$) δ 8.64 (d, *J* = 5.7 Hz, 4H), 7.58 (d, *J* = 8.5 Hz, 4H), 7.49 (d, *J* = 5.8 Hz, 4H), 7.43 (d, *J* = 8.7 Hz, 2H), 7.21 (d, *J* = 8.5 Hz, 4H), 7.06 (d, *J* = 8.7 Hz, 2H). ^{13}C NMR (100 MHz, $CDCl_3$) δ 150.26, 147.89, 147.41, 145.98, 132.86, 132.60, 128.05, 126.58, 124.23, 121.05, 116.73.

Synthesis of Compound **3**:

Two clean round-bottomed flutes were taken, and **2** (1.4 g, 2.9 mmol, 1.0 equiv.), bis(tributyltin) (1.87 g, 3.2 mmol, 1.1 equiv.) and tetrakis (triphenylphosphine) palladium (0) (167.6 mg, 0.15 mmol, 0.05 equiv.) were added successively. Under nitrogen protection, 30 mL of toluene was added for reflux reaction for 12 h. At the end of the reaction, the temperature was lowered to room temperature, and the crude product was separated by silica gel column chromatography (petroleum ether: ethyl acetate = 5:1, V/V) to obtain 611.5 mg yellow oily liquid **3** with a yield of 30%. ^{1}H NMR (400 MHz, $CDCl_3$) δ 8.64 – 8.62 (m, 4H), 7.57 (d, *J* = 8.7 Hz, 4H), 7.50 – 7.48 (m, 4H), 7.40 (d, *J* = 8.3 Hz, 2H), 7.24 – 7.21 (m, 4H), 7.14 (d, *J* = 8.3 Hz, 2H), 1.60 – 1.53 (m, 6H), 1.40 – 1.33 (m, 8H), 1.08 – 1.04 (m, 4H), 0.92 – 0.89 (m, 9H). ^{13}C NMR (101 MHz, $CDCl_3$) δ 150.26, 148.34, 147.55, 146.53, 137.57, 131.99, 128.45, 127.83, 124.81, 124.07, 120.97, 27.39.

Synthesis of Compound **5**:

Take a 50 mL round-bottled flask and add compound **3** (190 mg, 0.28 mmol, 20.0 equiv.), compound **4** (20.0 mg, 0.056 mmol, 1.0 equiv.) and trans-dichlorobis (triphenyl-phosphine) palladium (II) (4.0 mg, 0.005 mmol, 0.1 equiv.), and change the air in the flask three times with N_2. 15.0 mL of anhydrous THF was injected and then the mixture was stirred for 48 h at 100 °C. After being cooled to ambient temperature, the product was purified with column

chromatography (silica gel, DCM: MeOH=100: 1) to give a green solid compound **5** (19 mg, 35%).^{1}H NMR (400 MHz, $CDCl_3$) δ 8.64 (d, *J* = 5.7 Hz, 8H), 7.65 (d, *J* = 8.5 Hz, 2H), 7.60 (d, *J* = 8.6 Hz, 7H), 7.55 (d, *J* = 8.1 Hz, 3H), 7.50 (d, *J* = 5.7 Hz, 6H), 7.40 (d, *J* = 8.6 Hz, 4H), 7.26 (s, 10H). ^{13}C NMR (100 MHz, $CDCl_3$) δ 147.28, 145.14, 144.45, 142.95, 132.95, 124.93, 122.40, 122.17, 121.18, 118.11, 117.98.

Synthesis of Compound **6**:

To a 5 mL DCM solution of compound **5** (6.5 mg, 0.0065 mmol, 1.0 equiv.) was added trifluoromethanesulfonate (4.3 mg, 0.026 mmol, 4.0 equiv.), and the mixture was stirred at room temperature overnight. After the completion of the reaction, ether was added to the solution, and filter to give target **6** (5.5 mg, 80%). ^{1}H NMR (400 MHz, DMSO-d_6) δ 8.95 (t, *J* = 7.7 Hz, 8H), 8.46 (dd, *J* = 11.8, 6.8 Hz, 8H), 8.35 (d, *J* = 8.5 Hz, 4H), 8.15 (dd, *J* = 22.0, 8.7 Hz, 8H), 7.50 – 7.39 (m, 8H), 7.32 (dd, *J* = 13.4, 8.6 Hz, 4H), 4.32 (s, 12H). ^{13}C NMR (100 MHz, DMSO-d_6) δ 153.62, 152.56, 150.00, 145.87, 134.11, 130.31, 128.40, 125.87, 124.47, 123.53, 122.75, 47.35.

Fluorescence penetration depth measurement of BBTD-TPA NPs

Simulation of biological tissues with 1% intralipid emulsion for assessing the fluorescence penetration depth of **BBTD-TPA** NPs. Glass capillaries filled with **BBTD-TPA** NPs (200 μM) were fixed at the bottom of a cylindrical dish, followed by the addition of different volumes of 1% Intralipid mimic tissue depth. Finally, fluorescence images were obtained by using 808 nm laser illumination for **BBTD-TPA** NPs. Images were analyzed by ImageJ software.

Ultrasound irradiation penetration depth in mimic tissue

To assay the tissue depth response of **BBTD-TPA** NPs, agarose and Intralipid were used to simulate biological tissues. Briefly, 1 g of agarose was added to 100 mL of Intralipid (1%), and then the mixture was heated until the agarose was completely dissolved. Before the solution was completely solidified, the solution was injected into tubes at different depths to obtain simulated biological tissue with a variety of thicknesses. After tissue models were obtained, the models were placed on top of the ultrasound instrument probe, followed by placing **BBTD-TPA** NPs (20 μM) containing 20 μM DCFH-DA on top of the different height models. After US irradiation for 1 min (1.0 MHz, 0.5 W cm^{-2}, 50% duty cycle), the mixture was transferred to a 96-well plate, and ROS fluorescence images were measured using Amersham Thphoon 5.

LC3 immunofluorescence staining

BV2 cells were seeded into confocal dishes and cultured overnight. Following the treatment with **BBTD-TPA** NPs and ultrasound, the cells were rinsed three times with sterile PBS and subsequently permeabilized with 0.1% Triton X-100 in PBS for a period of five minutes. This was followed by a 30-minute incubation in a confinement solution. Subsequently, the cells were incubated at 4 °C overnight with anti-LC3B antibody (ab192890), after which they were rinsed with PBS and incubated with CoraLite488-conjugate secondary antibody (SA00013-2) for 1 h at room temperature. Thereafter, the cells were imaged using confocal microscopy and the images were analyzed by image J.

Intracellular ROS measurement

BBTD-TPA NPs (40 μM) were incubated with BV2 cells for 12 h. Subsequently, the DCFH-DA fluorescent probe (20 μM) was added and incubated in the dark for 30 min. Then the BV2 cells were exposed to US irradiation (1.0 MHz, 0.5 W cm^{-2}, 50% duty cycle). Following two rinses with PBS, *in vitro* fluorescence imaging was conducted using an inverted fluorescence microscope.

JC-1 fluorescence staining

BV2 cells (3×10^5 cells/well) were seeded in confocal dishes overnight, and **BBTD-TPA** NPs (50 μM, 500 μL) were coincubated with BV2 cells for 12 h. Then, the US and SDT group was exposed to US irradiation (0.5 W cm^{-2}, 1 MHz, 50% duty cycle, 1 min) and continue growth 12 h. After washing with PBS, the BV2 cells were coincubated with fresh MEM containing the JC-1 probe (2 μg/mL) at 37 °C for 10 min. After washing twice with PBS, fluorescence imaging was observed with a confocal laser scanning microscope. The images were analyzed by ImageJ software.

Mitochondrial colocalization study

BV2 cells (1×10^4) were seeded in confocal dishes and incubated overnight. The cells were then incubated with Mito-Tracker Green (C1048, Beyotime Biotechnology, China) for 45 min. After rinsing the cells twice with PBS, the cells were observed under a NIR-II inverted fluorescence microscope. The excitation wavelength of Mito-Tracker Green was 490 nm, with an emission wavelength of 516 nm. The images were analyzed using ImageJ software.

Transmission electron microscope (TEM)

After digesting the treated cells, centrifuge them twice with PBS and add 2.5% glutaraldehyde solution and fixed at room temperature away from light for 1 h, they were washed twice with PBS again. The samples are embedded in Epon after they have been dehydrated with ethanol in graded amounts. In this study, ultra-thin slices were stained with UO2 acetate and lead citrate at a concentration of 2%. Observations were conducted under a transmission electron microscope.

Hemolysis Assay

To evaluate the hemolysis of **BBTD-TPA** NPs, fresh blood from BALB/c mice was saved in heparinized tubes and then washed three times with PBS to obtain an RBC solution. Next, 0.5 mL RBC solution was added to 0.5 mL **BBTD-TPA** NPs (dissolved in PBS) at different concentrations. The mixture was incubated at 37 °C for 60 min. Then, the mixture was centrifuged at 3000 rpm for 10 min to remove sediment. After pictures were taken, the supernatant (100 μL) was plated in 96-well plates to record the OD value at 540 nm. In addition, saline was used as a negative control, while water was used as a positive control. The hemolysis rate was calculated by using the following equation:

$$\text{Hemolysis Rate (\%)} = (OD_{sample} - OD_{saline})/(OD_{water} - OD_{saline}) \times 100\%$$

Tissue-level ROS detection *in vivo*

Intracellular ROS generated by ultrasound-activated **BBTD-TPA** NPs were detected by DHE fluorescent dyes. PVN sections were dried, circles were drawn around the tissue with a

histochemistry pen, an autofluorescence quencher was added for 5 min, rinsed with running water, DHE staining solution was added dropwise to the circles, and incubated for 30 min at 37 °C in a light-proof thermostat after three washes with PBS. An inverted fluorescence microscope was used for fluorescence imaging, and images were captured with a scanner (Pannoramic MIDI, 3DHISTECH, Budapest, Hungary).

Enzyme-linked immunosorbent assay (ELISA)

Blood samples were obtained by puncture from the right ventricle, followed by centrifugation at 3000 rpm for 15 min and removal of the upper serum layer. Serum levels of IL-1β (GER0002, Servicebio, China) and IL-6 (GER0001, Servicebio, China) were detected by ELISA following the manufacturers' instructions.

TTC staining

Following the euthanasia of the rats, the hearts were removed and rapidly transferred to a -40°C refrigerator to be frozen for 15 min. The hearts were then sliced into 5 mm-thick slices and placed in a 37°C incubator for 10 min. Subsequently, the slices were washed with phosphate-buffered saline (PBS) and immersed in 4% paraformaldehyde fixative for 24 h [1, 2]. Images were acquired using a white light camera and analyzed using Image Pro Plus 6.0 software.

Measurement of ERP

The ERP was measured at the left ventricular apex, middle, and bottom of the myocardium and determined by the programmed stimulus, which consisted of 8 continuous stimuli (S1-S1, 150 ms cycle length) followed by a premature stimulus (S2). The S1-S2 interval was initially reduced by 10 ms from 150 ms to the nonresponse period. The ERP is the longest S1-S2 interval that fails to capture the ventricle [3, 4].

References

1. Zhou L, Zhang Y, Cao G, Zhang C, Zheng C, Meng G, et al. Wireless self-powered optogenetic system for long-term cardiac neuromodulation to improve post-MI cardiac remodeling and malignant arrhythmia. Adv Sci. 2023; 10:e2205551.
2. Yu Z, Han J, Chen H, Wang Y, Zhou L, Wang M, et al. Oral supplementation with butyrate improves myocardial ischemia/reperfusion injury via a gut-brain neural circuit. Front Cardiovasc Med. 2021; 8: 718674.
3. Wang S, Wu L, Li X, Li B, Zhai Y, Zhao D, et al. Light-emitting diode therapy protects against ventricular arrhythmias by neuro-immune modulation in myocardial ischemia and reperfusion rat model. J Neuroinflammation. 2019; 16:139.
4. Yu L, Zhou L, Cao G, Po SS, Huang B, Zhou X, et al. Optogenetic modulation of cardiac sympathetic nerve activity to prevent ventricular arrhythmias. J Am Coll Cardiol. 2017; 70:2778-2790.

2. Supplementary Figures

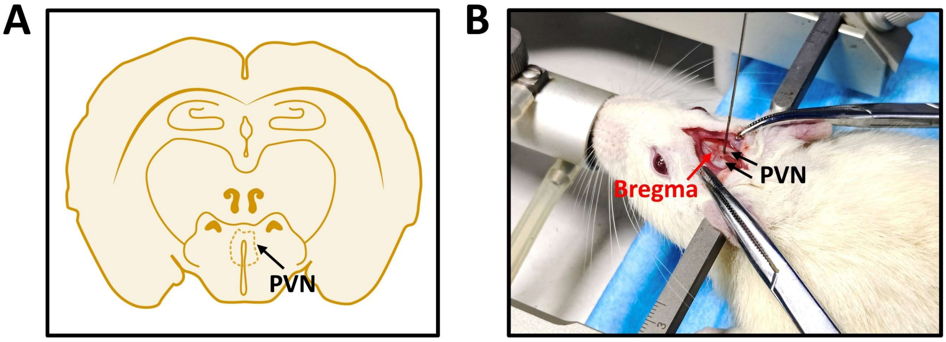

Figure S1. A) Schematic diagram of the position of the PVN. B) PVN localization and microinjection according to bregma.

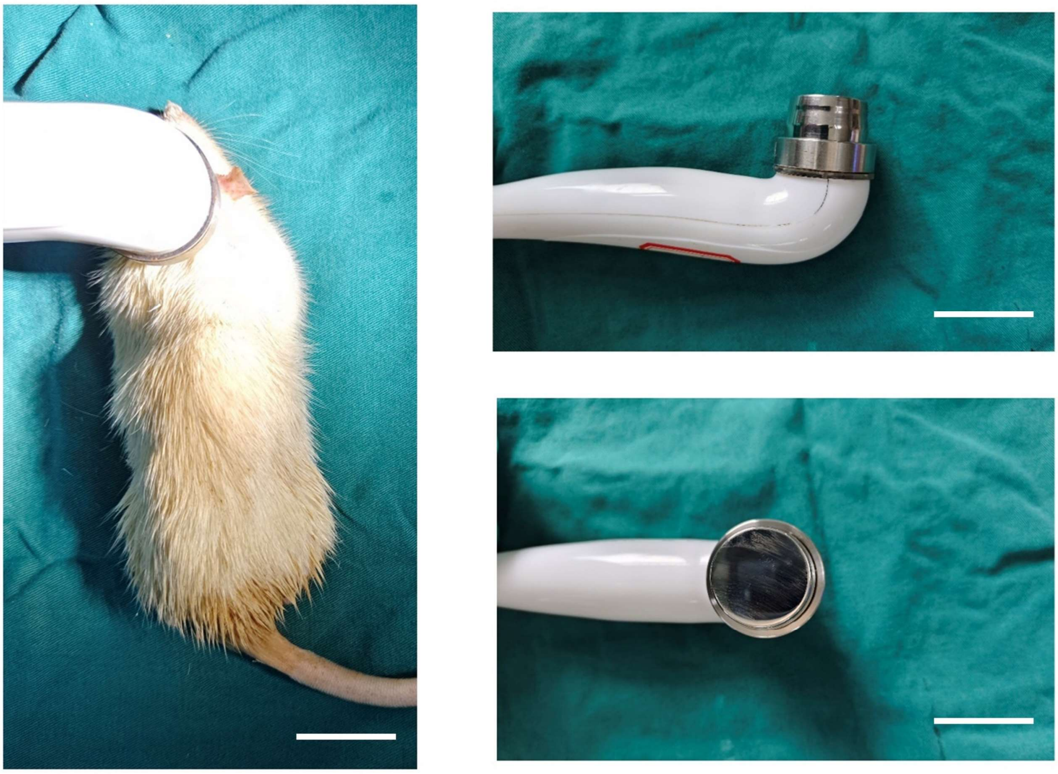

Figure S2. Diagrams of ultrasound probe and rats undergoing sonodynamic therapy. Scale bar = 25 mm.

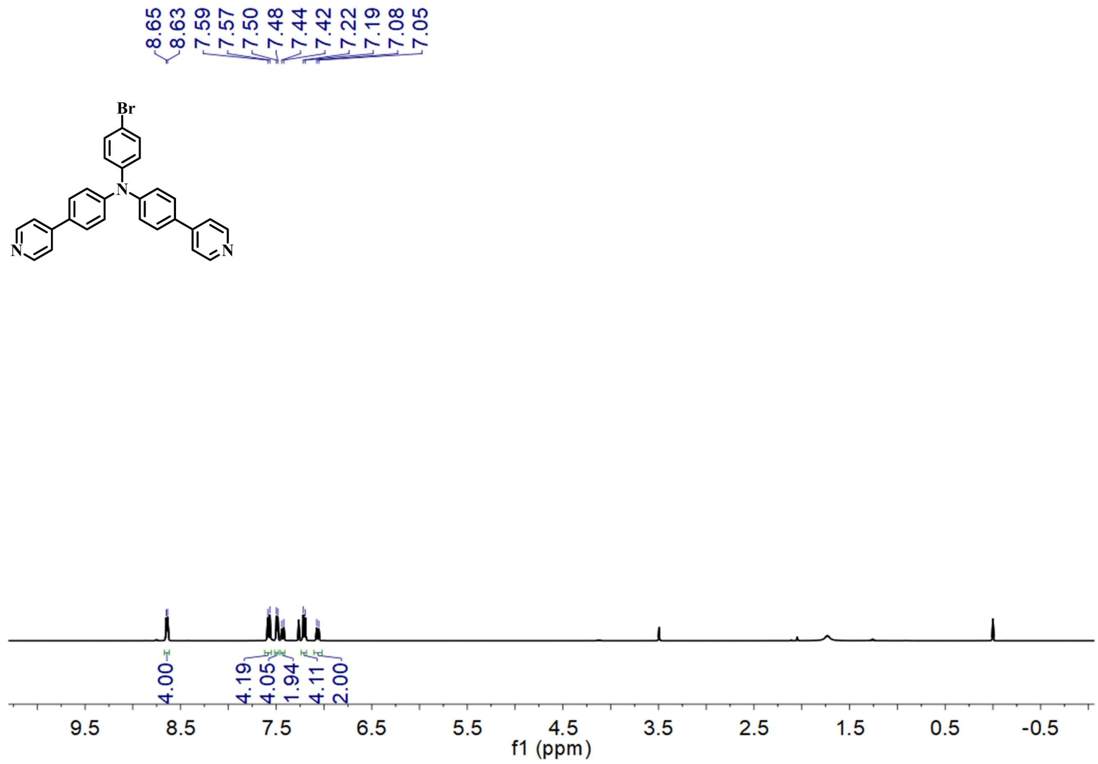

Figure S3. ^{1}H NMR spectrum (400 MHz, CDCl3, 298 K) of Compound **2**.

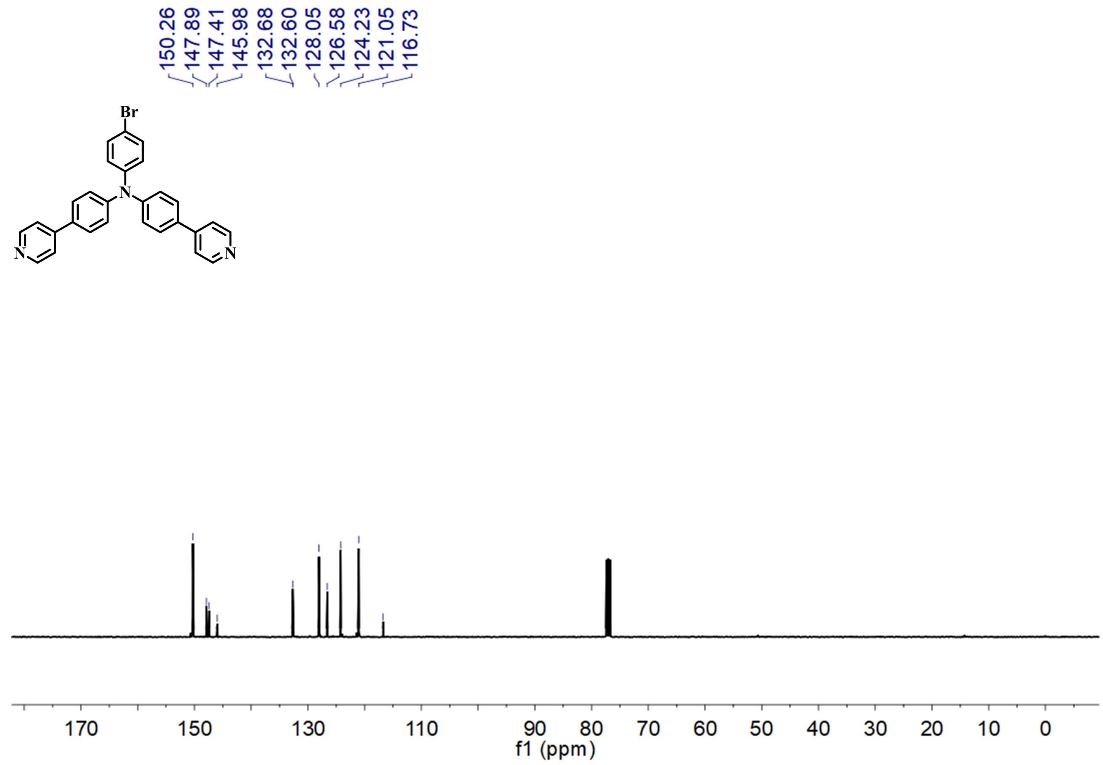

Figure S4. ^{13}C NMR spectrum (400 MHz, CDCl3, 298 K) of Compound **2**.

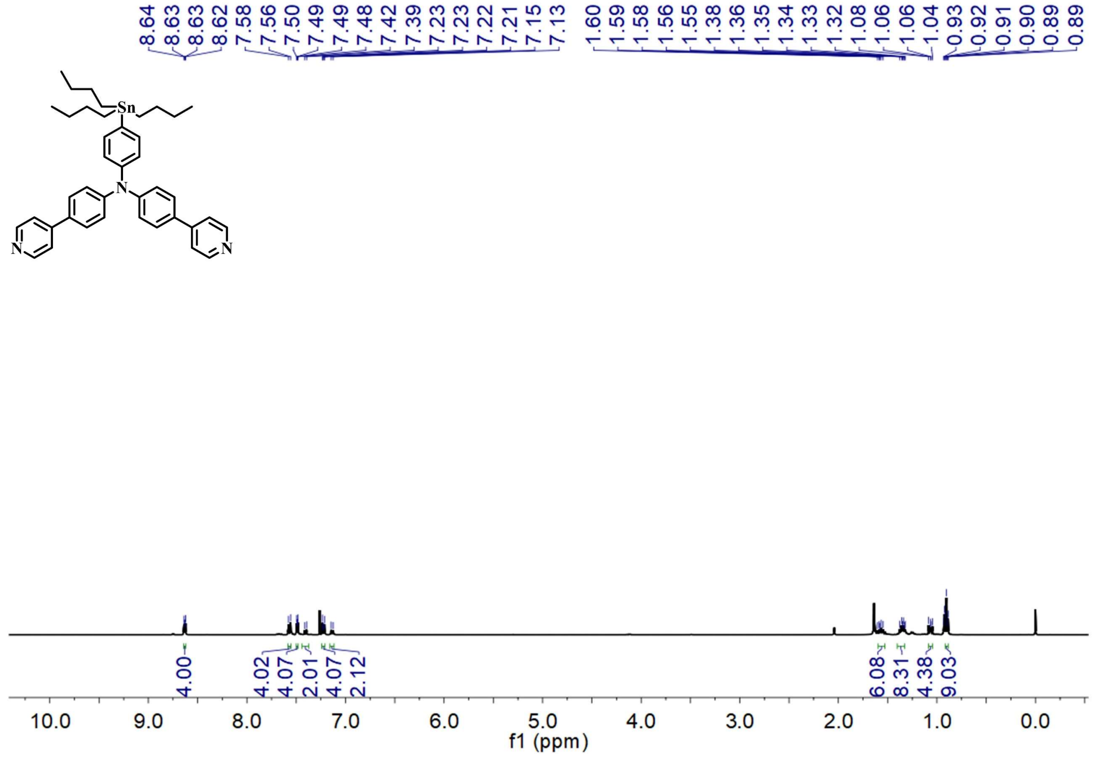

Figure S5. ^{1}H NMR spectrum (400 MHz, CDCl3, 298 K) of Compound **3**.

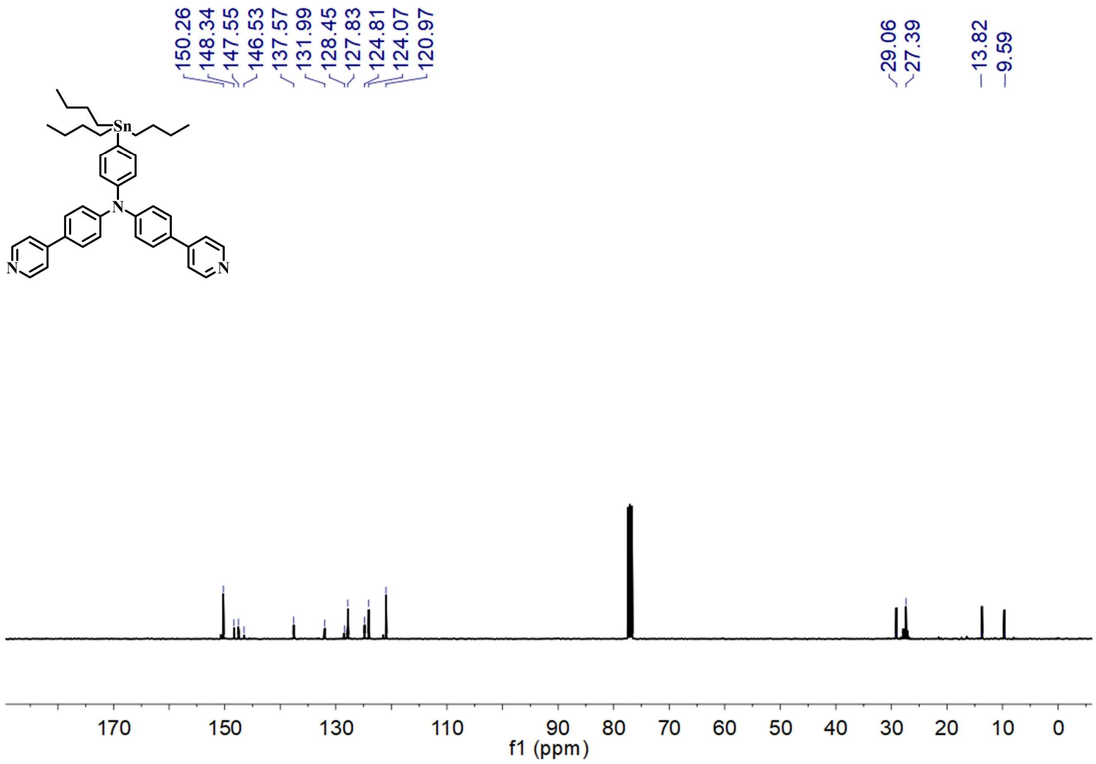

Figure S6. ^{13}C NMR spectrum (400 MHz, CDCl3, 298 K) of Compound **3**.

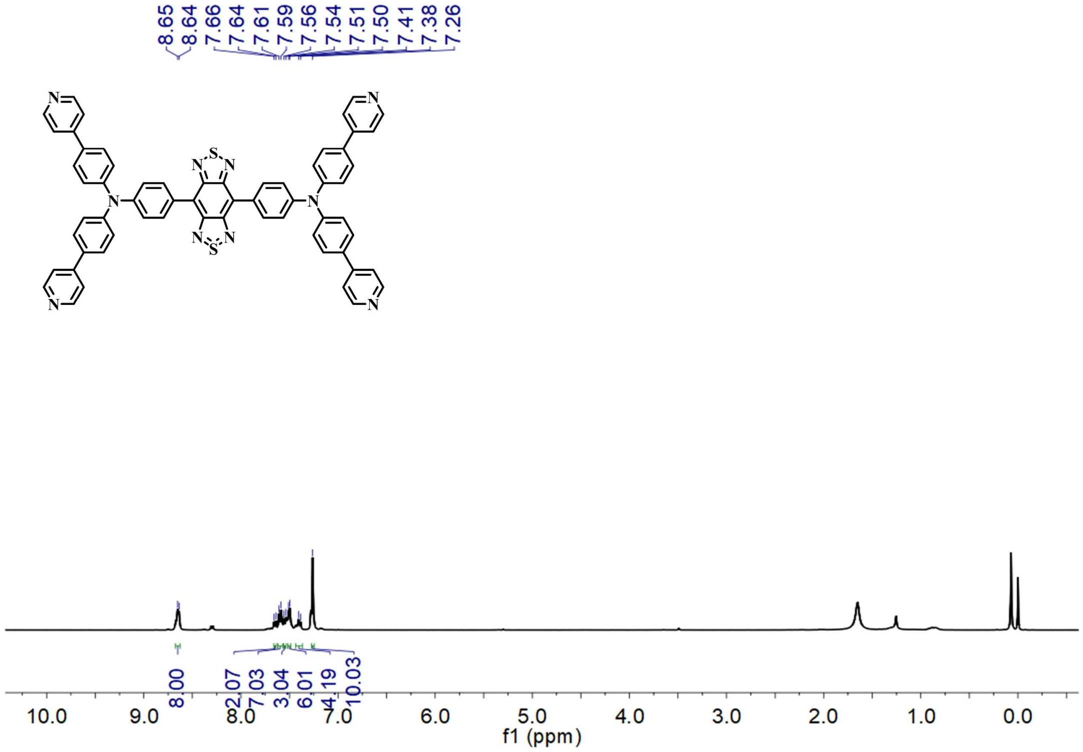

Figure S7. ^{1}H NMR spectrum (400 MHz, CDCl3, 298 K) of Compound **5**.

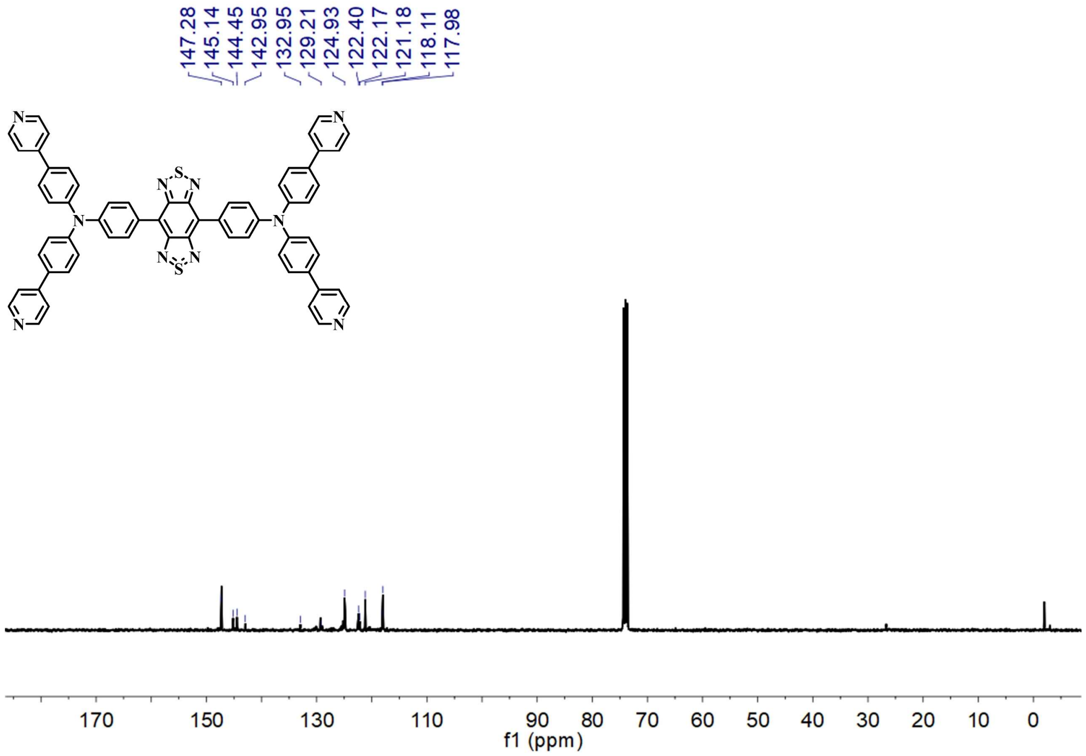

Figure S8. ^{13}C NMR spectrum (400 MHz, CDCl3, 298 K) of Compound **5**.

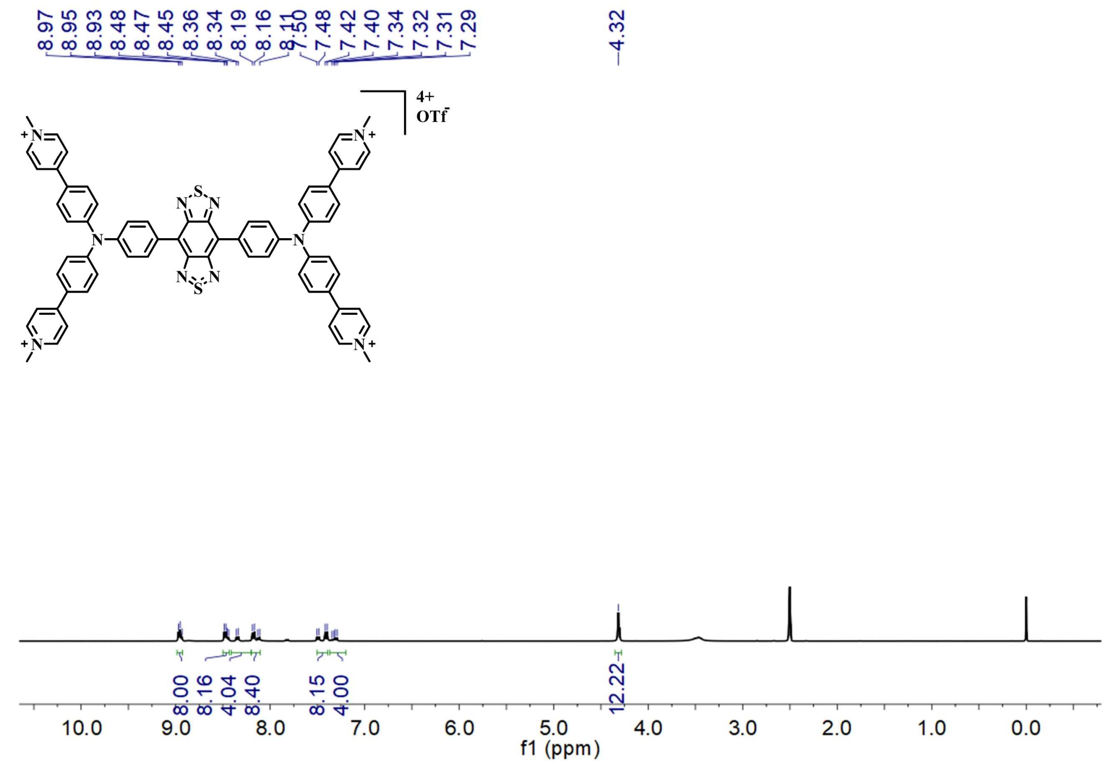

Figure S9. ^{1}H NMR spectrum (400 MHz, DMSO-d_6, 298 K) of Compound **6**.

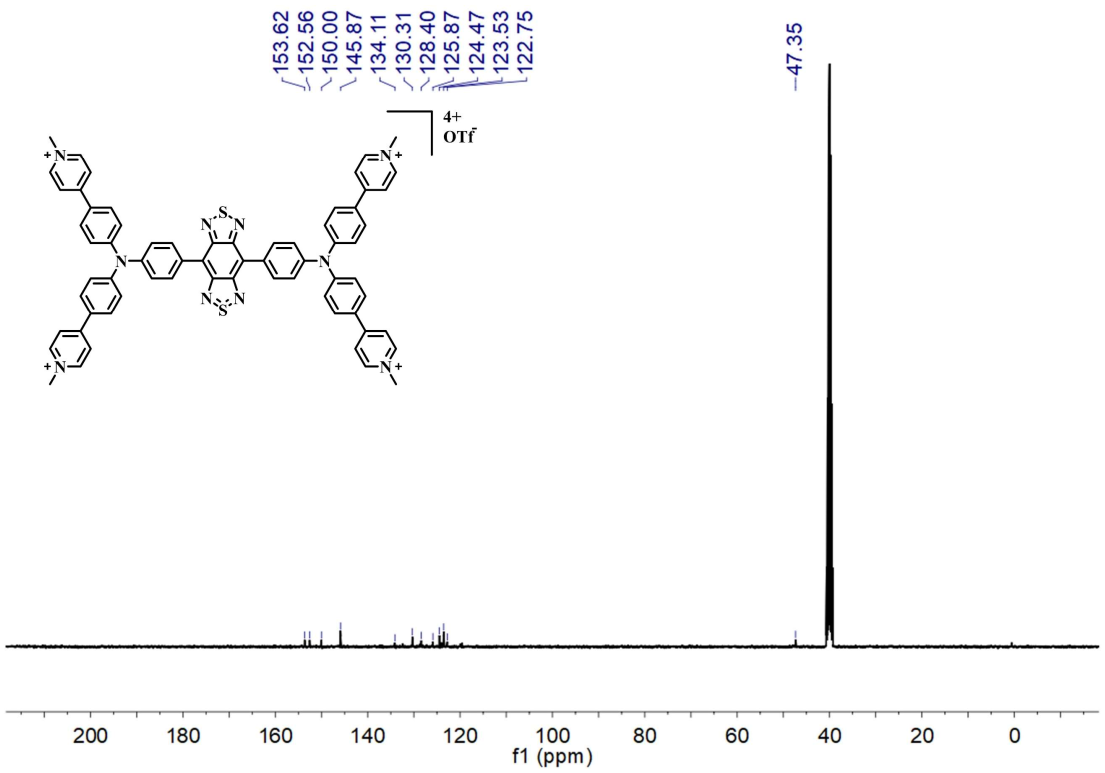

Figure S10. ^{13}C NMR spectrum (400 MHz, DMSO-d_6, 298 K) of Compound **6**.

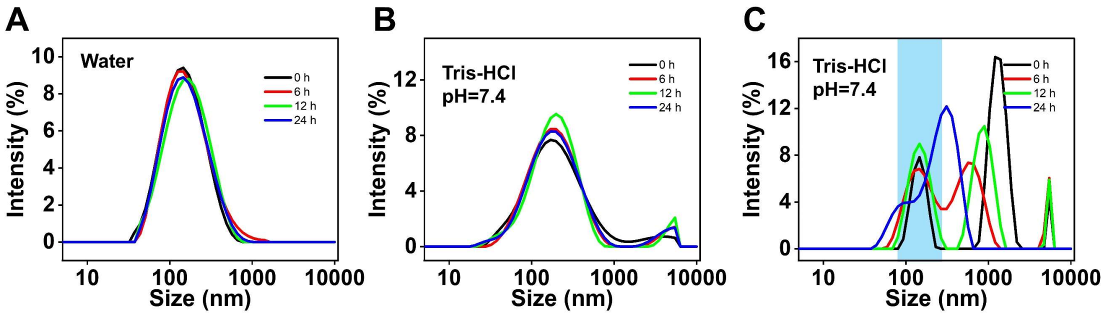

Figure S11. Size distribution of **BBTD-TPA** NPs under the conditions of A) water (pH 7.0), B) Tris-HCl (pH 7.4), C) Tris-HCl (pH 5.0).

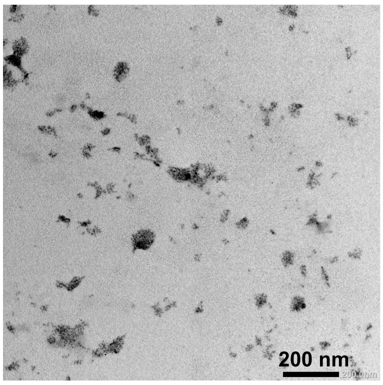

Figure S12.TEM morphology of **BBTD-TPA** NPs after 12 h of incubation with Tris-HCl (pH 5.0). Scale bar = 200 nm.

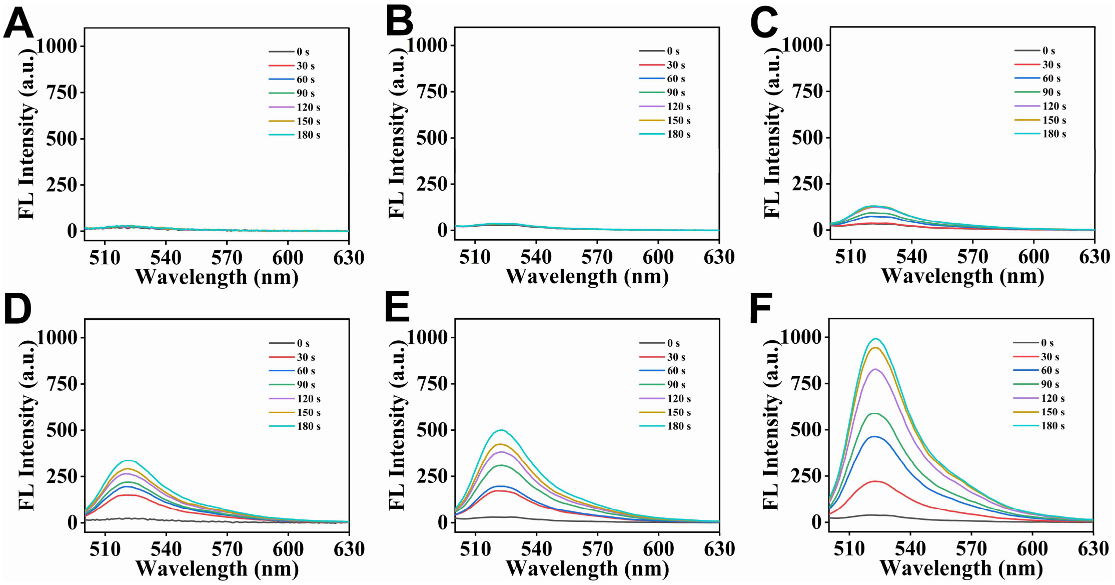

Figure S13. ROS production of **BBTD-TPA** NPs (20 μM) under various US power (0, 0.5, 1.0, 1.5, 2.0 and 2.5 W cm^{-2}) (indicator: DCFH-DA).

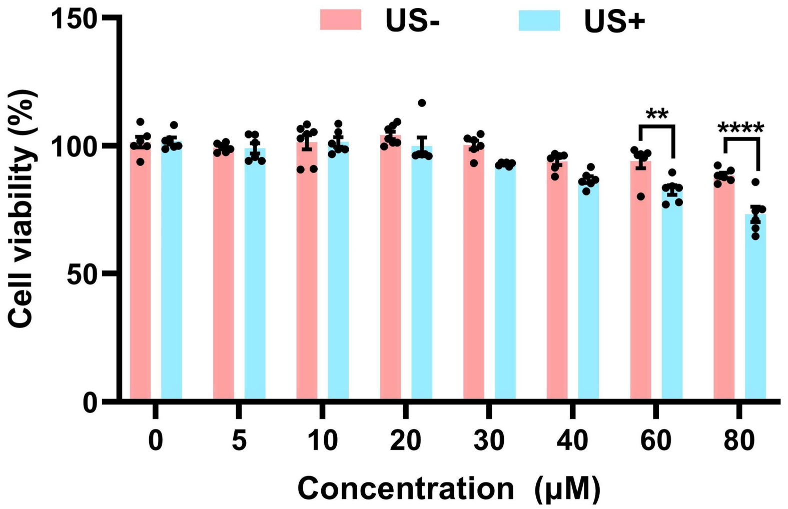

Figure S14. The relative cell viability of BV2 cells treated with various concentrations of **BBTD-TPA** NPs with or without US irradiation (n = 6 per group). Data are expressed as mean ± SEM, analyzed by unpaired t-test. **$P<0.01$, and ****$P<0.0001$ versus US- group.

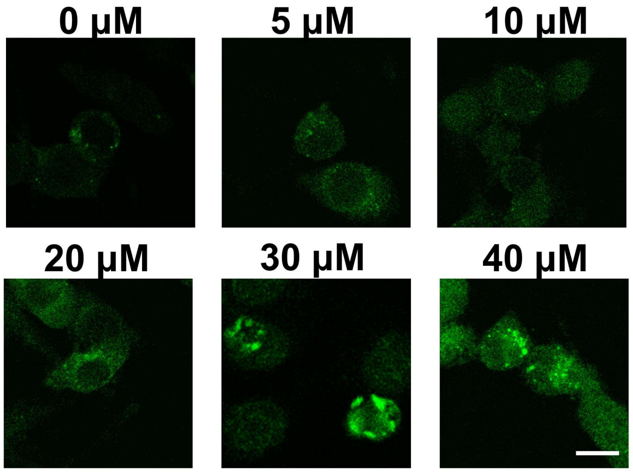

Figure S15. Representative images of LC3 immunofluorescence staining in BV2 cells treated with various concentrations of **BBTD-TPA** NPs with ultrasound irradiation. Scale bar = 10 μm.

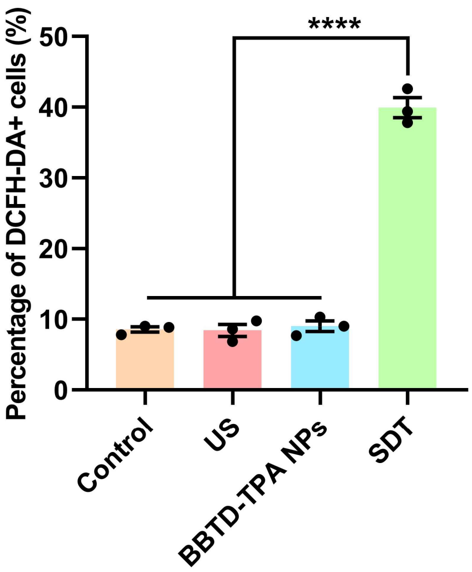

Figure S16. Statistical analysis of the percentage of DCFH-DA+ cells by flow cytometry. ****$P<0.0001$.

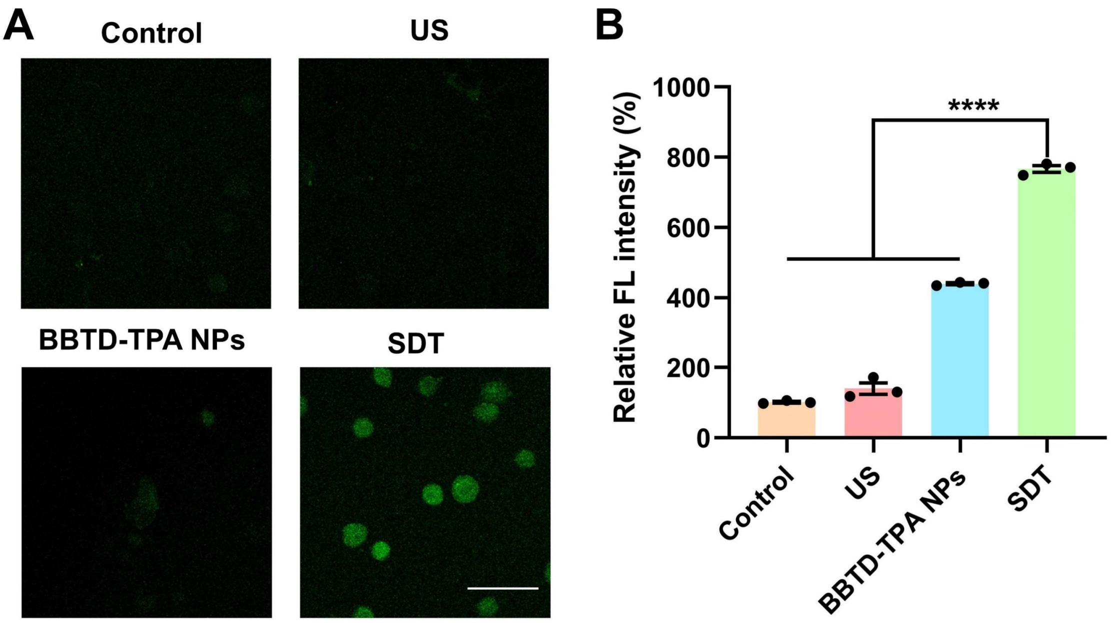

Figure S17. A) Confocal laser scanning microscope (CLSM) images of intracellular ROS detection in differently treated BV2 cells stained with DCFH-DA. Scale bar = 50 μm. B) Statistical analysis of DCFH-DA fluorescence intensity. ****$P<0.0001$.

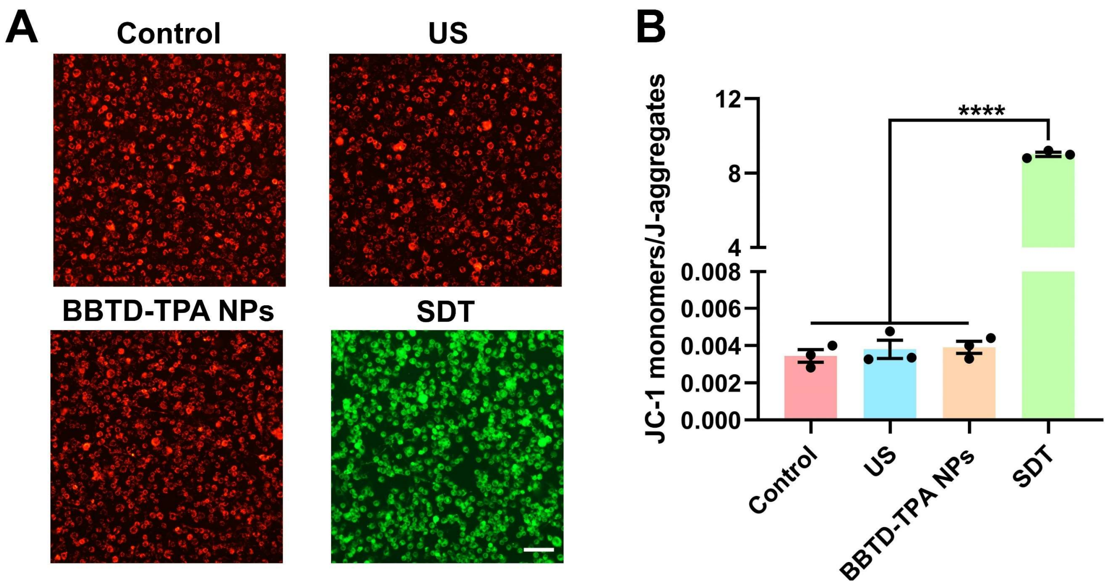

Figure S18. A) Typical images of JC-1 staining in BV2 cells. Green channel, JC-1 monomers; red channel, J-aggregates. Scale bar = 100 μm. B) Quantitative analysis of the JC-1 fluorescence intensity (n=3 per group). $^{****}P<0.0001$.

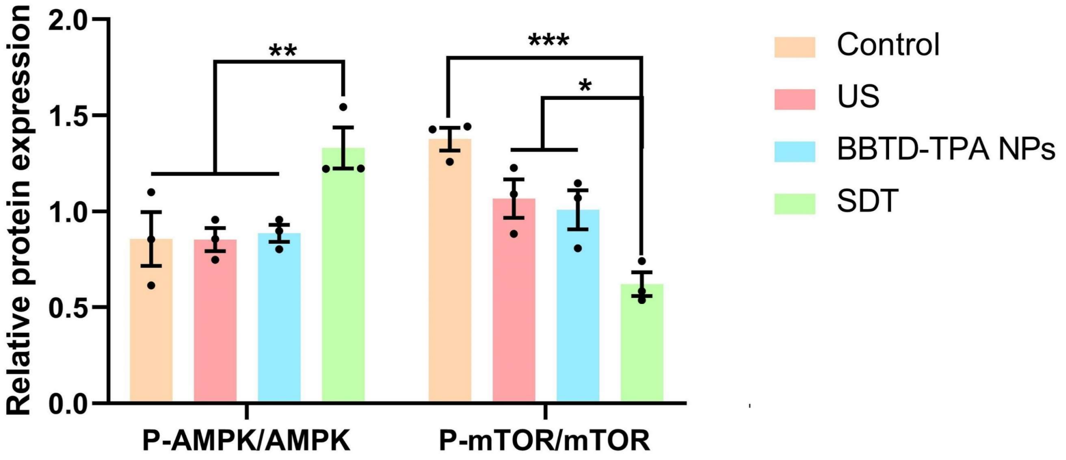

Figure S19. Quantitative analysis of the protein levels of P-AMPK/AMPK, and P-mTOR/mTOR after different treatments (n = 3 per group). $^{*}P<0.05$, $^{**}P<0.01$, and $^{***}P<0.001$.

Full uncropped blot for Figure 3A

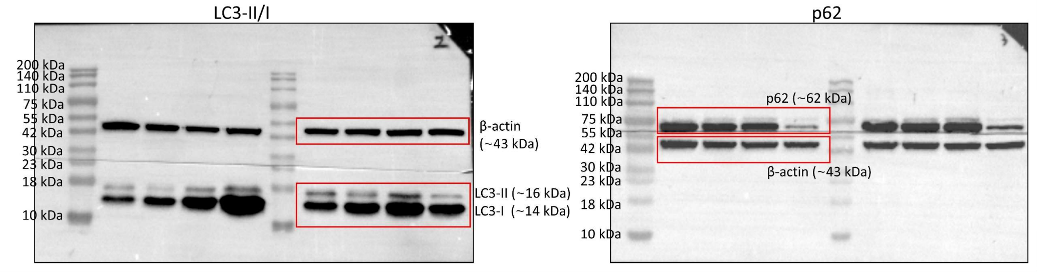

Full uncropped blot for Figure 3G

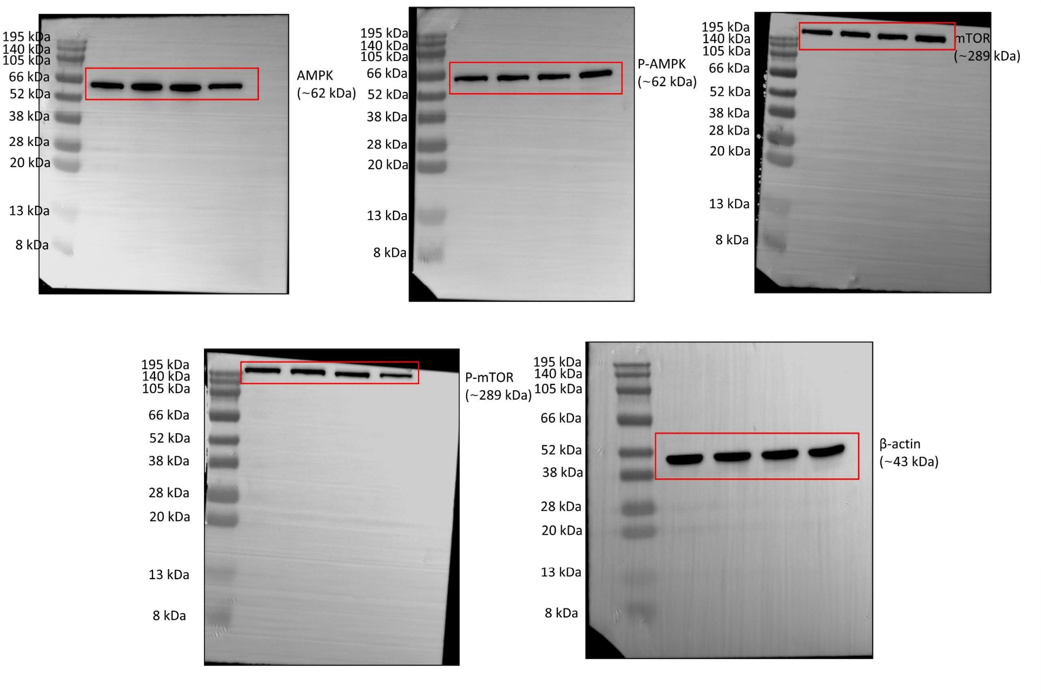

Figure S20. Full uncropped gels for Western blot for Figure 3.

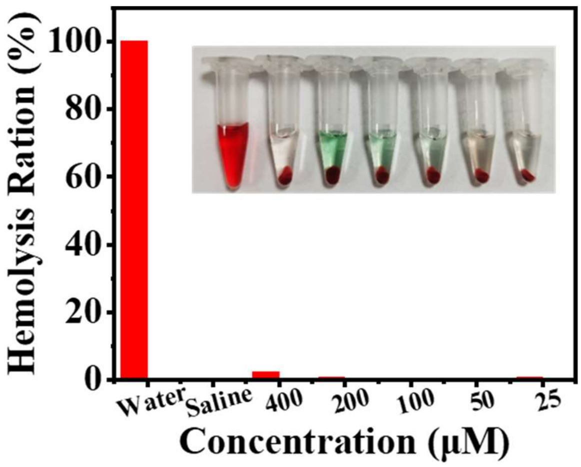

Figure S21. Hemolysis tests of **BBTD-TPA** NPs at various concentration (25, 50, 100, 200, and 400 μM). The insert was the optical photograph.

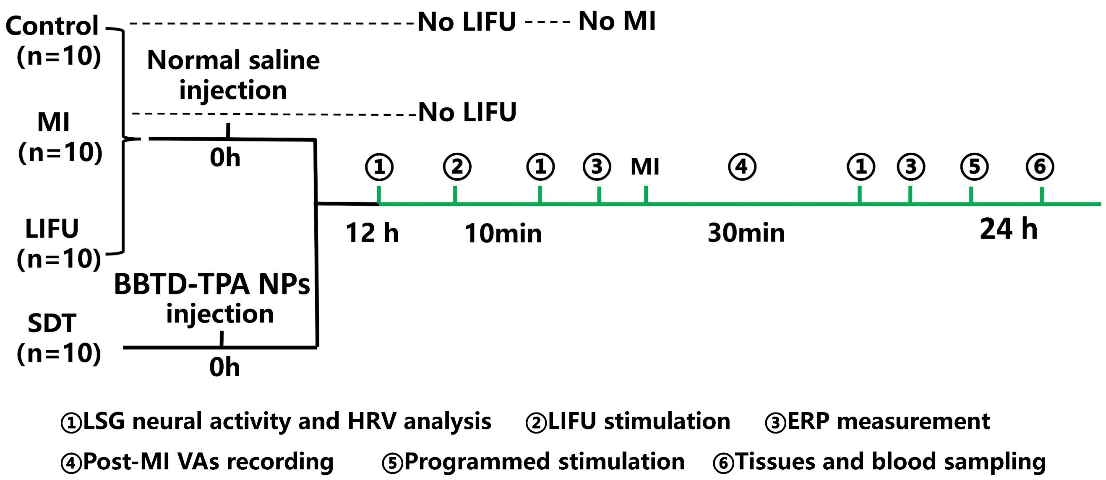

Figure S22. Flowchart of the experimental design *in vivo*.

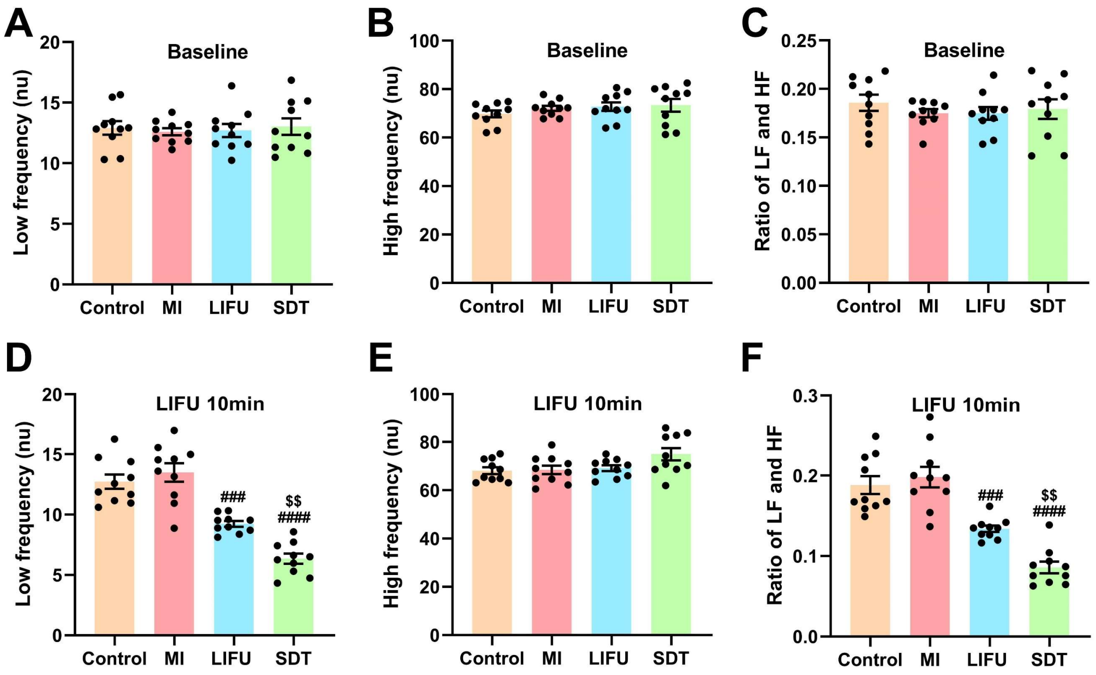

Figure S23. HRV analysis of LF, HF, and LF/HF ratio in the four groups at baseline and after LIFU 10 min (n=10 per group). Data are expressed as mean ± SEM. ###*P<0.001* and ####*P<0.0001* versus the MI group; $$*P<0.01* versus the LIFU group. LF, low frequency; HF, high frequency.

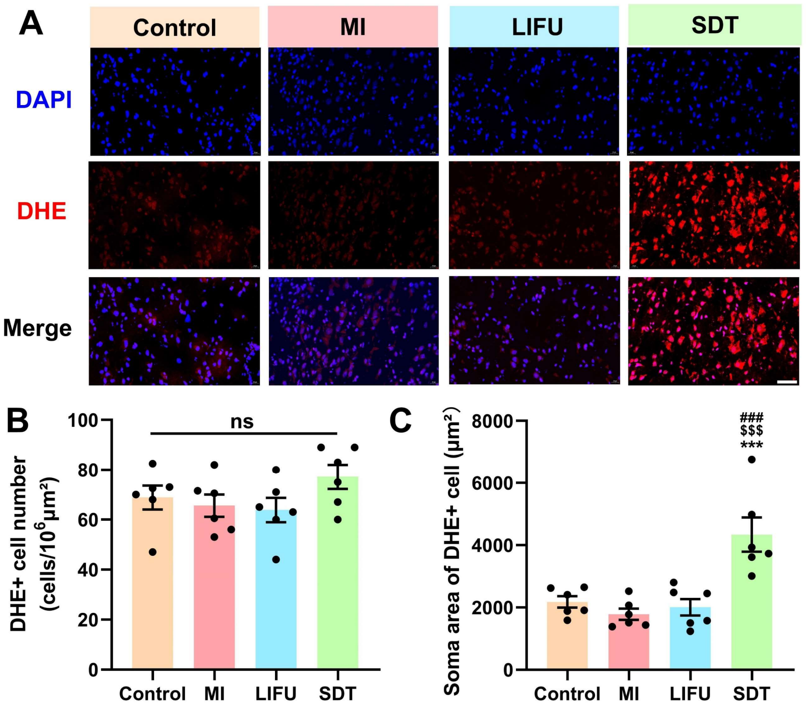

Figure S24. A) Typical images of DHE staining in the PVN in different groups. Scale bar = 100 μm. B, C) Quantitative analysis of the numbers and soma area of DHE+ cells (n=6 per group). Data are expressed as mean ± SEM. ****P<0.001* versus the control group; $^{\#\#\#}$*P<0.001* versus the MI group; $^{\$\$\$}$*P<0.001* versus the LIFU group; ns, no significance.

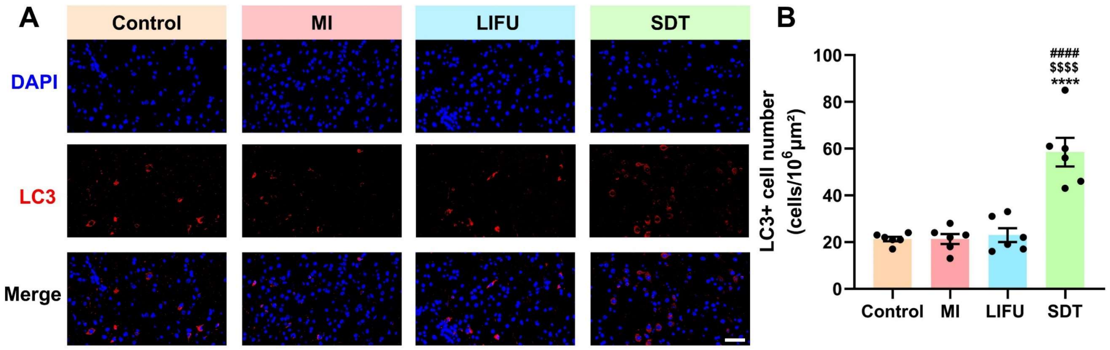

Figure S25. A) Representative images of LC3 immunofluorescence staining within the PVN. Scale bar = 100 μm. B) Quantitative analysis of LC3+ cell number (n=6 per group). Data are expressed as mean ± SEM. *****P<0.0001* versus the control group; $^{\#\#\#\#}$*P<0.0001* versus the MI group; $^{\$\$\$\$}$*P<0.0001* versus the LIFU group.

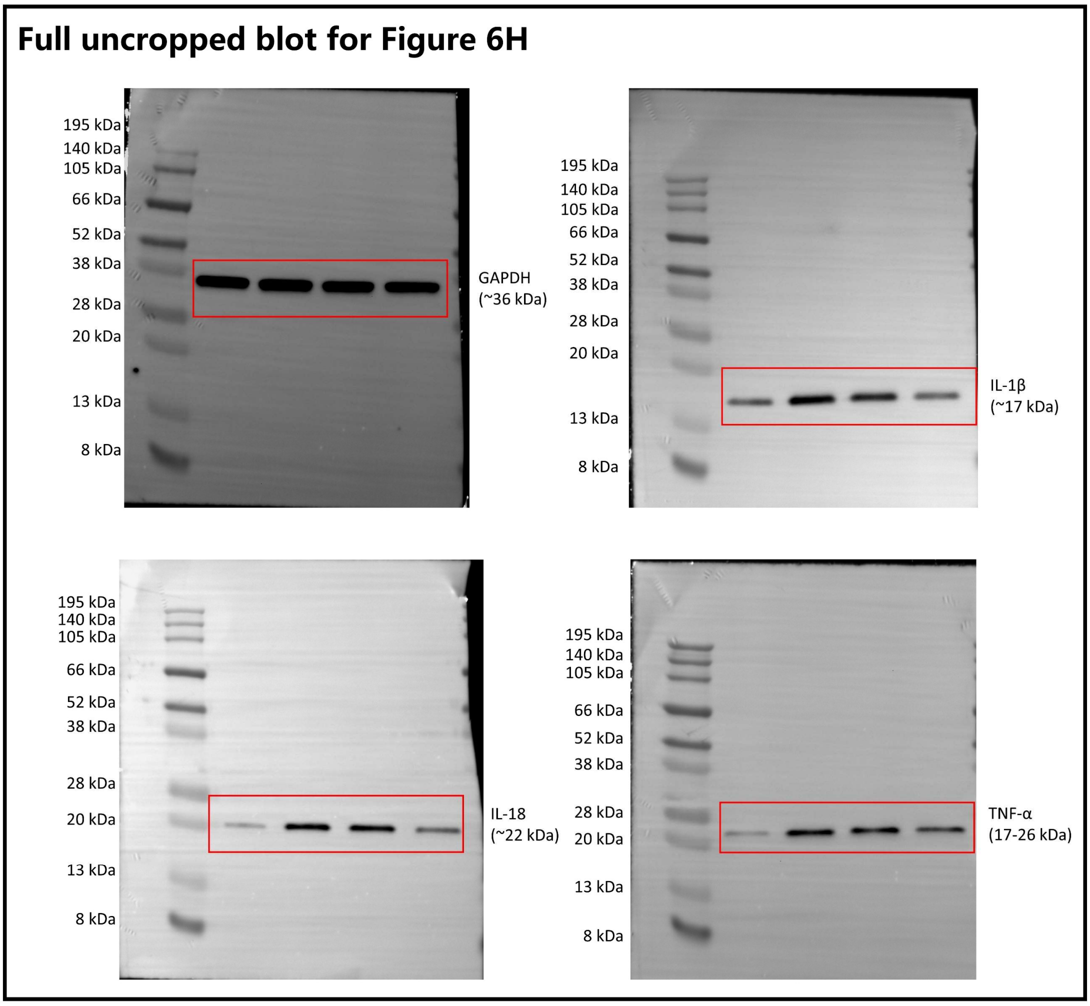

Figure S26. Full uncropped gels for Western blot for Figure 6.

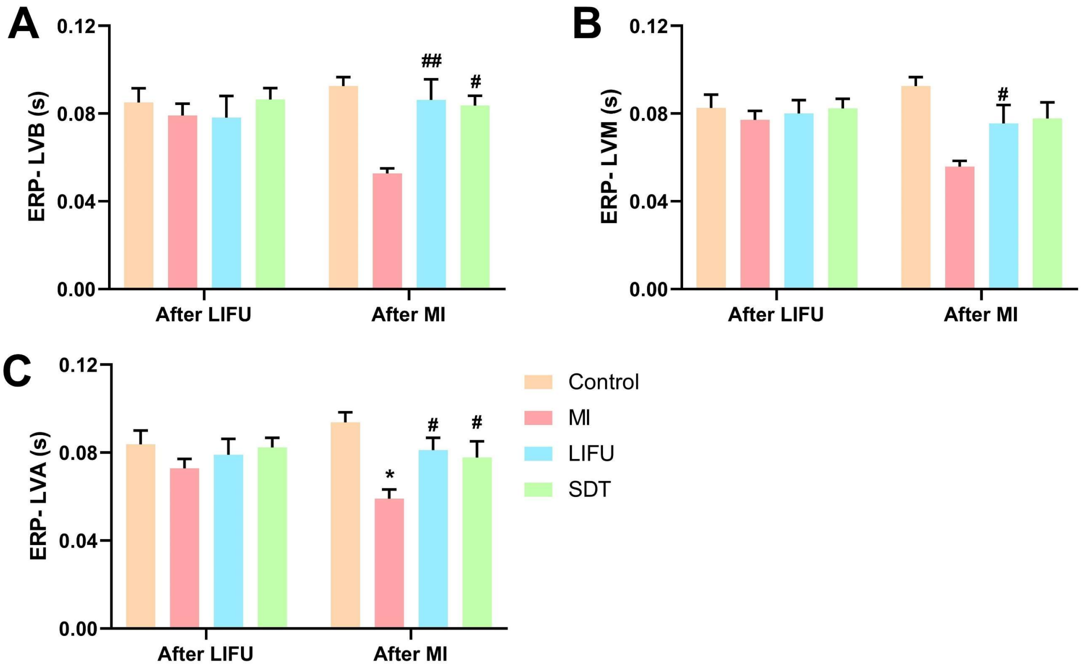

Figure S27. ERP of distinct regions of the ventricle after LIFU and MI (n=8-10 animals per group). Data are expressed as mean ± SEM. **P<0.05* versus the control group; $^{\#}$*P<0.05* and $^{\#\#}$*P<0.01* versus the MI group. ERP, effective refractory period; LVB, left ventricular bottom; LVM, left ventricular middle; LVA, left ventricular apex.

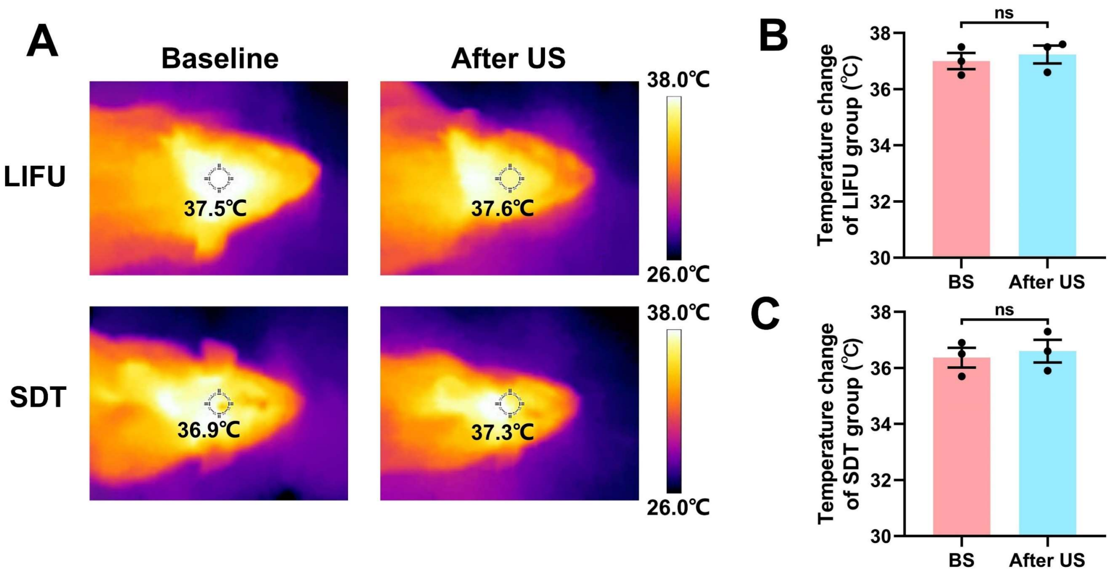

Figure S28. Biosafety assessment of **BBTD-TPA** NPs-mediated SDT. A) Thermal images of PVN before and after US treatment. No apparent temperature changes were observed in the B) LIFU and C) SDT groups (n=3 per group). Data are expressed as mean ± SEM. BS, baseline; US, ultrasound; ns, no significance.

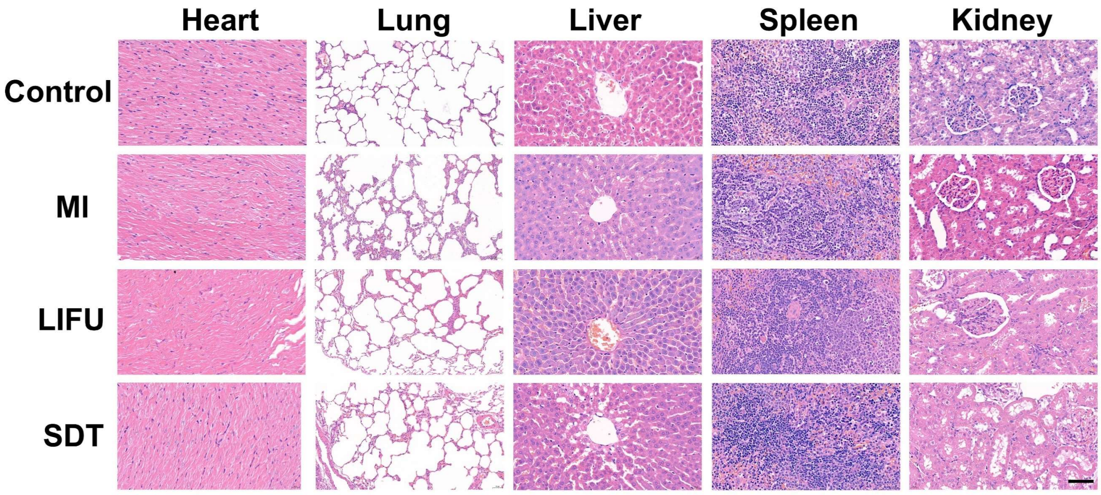

Figure S29. H&E staining of heart, lung, liver, spleen and kidney in different groups. Scale bar = 100 μm.

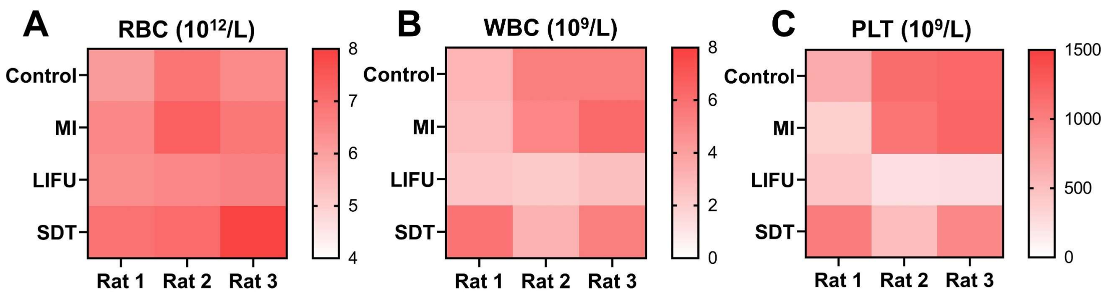

Figure S30. Blood cell counts in different groups (n=3 animals per group). RBC, red blood cell (5-9.8); WBC, white blood cell (1.9-16.8); PLT, platelet (250-1500). Normal range is indicated in brackets and all values are within this range.

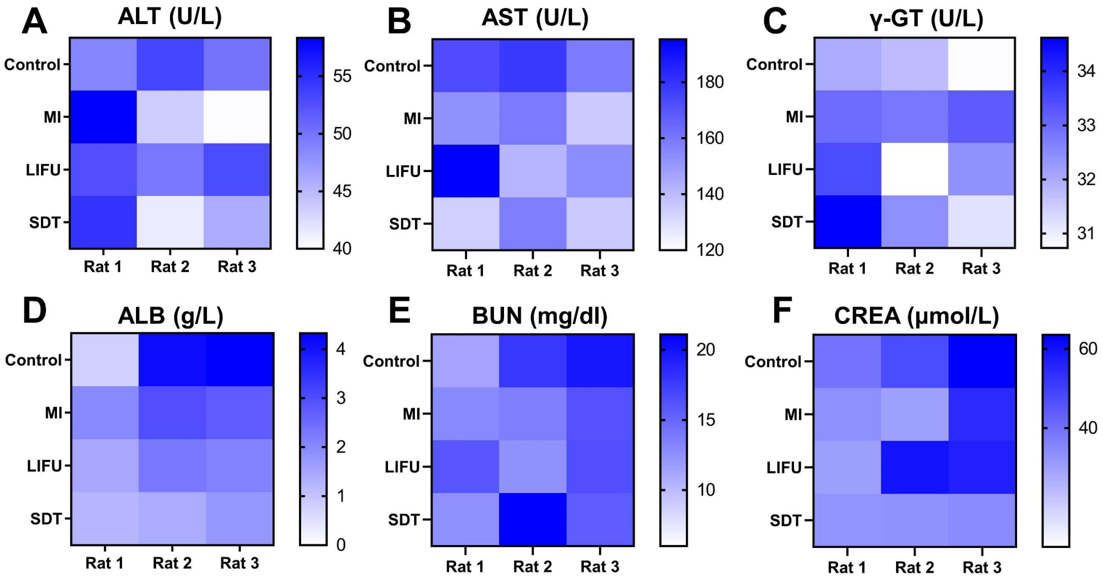

Figure S31. Biochemical indicators tests in various group (n=3 per group). ALT, alanine aminotransferase (21.53-61.75 U/L); AST, glutamic oxaloacetic transaminase (41.47-195.65 U/L); γ-GT, γ-glutamyltransferase (0.58-6.81 U/L); ALB, albumin (21.16-34.77 g/L); BUN, blood urea nitrogen (9.75-22.71 mg/dl); CREA, creatinine (10.90-118.07 μmol/L). Normal range is indicated in brackets and all values are within this range.
